# Radioresistance of mesenchymal glioblastoma initiating cells correlates with patient outcome and is associated with activation of inflammatory program

**DOI:** 10.18632/oncotarget.18363

**Published:** 2017-06-03

**Authors:** Elisabetta Stanzani, Fina Martínez-Soler, Teresa Martín Mateos, Noemi Vidal, Alberto Villanueva, Miquel Angel Pujana, Jordi Serra-Musach, Núria de la Iglesia, Pepita Giménez-Bonafé, Avelina Tortosa

**Affiliations:** ^1^ Department of Physiological Sciences, Faculty of Medicine and Health Sciences, Universitat de Barcelona, IDIBELL, L’Hospitalet del Llobregat, Barcelona, Spain; ^2^ Department of Basic Nursing, Faculty of Medicine and Health Sciences, Universitat de Barcelona, IDIBELL, L’Hospitalet del Llobregat, Barcelona, Spain; ^3^ Department of Pathology, Hospital Universitari de Bellvitge, IDIBELL, L’Hospitalet del Llobregat, Barcelona, Spain; ^4^ Program Against Cancer Therapeutic Resistance (ProCURE), Catalan Institute of Oncology, IDIBELL L’Hospitalet del Llobregat, Barcelona, Spain; ^5^ Xenopat S.L., Bellvitge Health Science Campus, L’Hospitalet del Llobregat, Barcelona, Spain; ^6^ Glioma and Neural Stem Cell Group, August Pi i Sunyer Biomedical Research Institute (IDIBAPS), Barcelona, Spain

**Keywords:** glioblastoma, cancer stem cells, radiotherapy, radioresistance, inflammation

## Abstract

Glioblastoma (GBM) still remains an incurable disease being radiotherapy (RT) the mainstay treatment. Glioblastoma intra-tumoral heterogeneity and Glioblastoma-Initiating Cells (GICs) challenge the design of effective therapies. We investigated GICs and non-GICs response to RT in a paired *in-vitro* model and addressed molecular programs activated in GICs after RT. Established GICs heterogeneously expressed several GICs markers and displayed a mesenchymal signature. Upon fractionated RT, GICs reported higher radioresistance compared to non-GICs and showed lower α- and β-values, according to the Linear Quadratic Model interpretation of the survival curves. Moreover, a significant correlation was observed between GICs radiosensitivity and patient disease-free survival. Transcriptome analysis of GICs after acquisition of a radioresistant phenotype reported significant activation of Proneural-to-Mesenchymal transition (PMT) and pro-inflammatory pathways, being STAT3 and IL6 the major players. Our findings support a leading role of mesenchymal GICs in defining patient response to RT and provide the grounds for targeted therapies based on the blockade of inflammatory pathways to overcome GBM radioresistance.

## INTRODUCTION

Glioblastoma (GBM) is the most common malignant type of adult primary brain tumor and is characterized by extreme therapeutic resistance that leads to poor patient outcome [1]. Radiation therapy (RT) has been the cornerstone of GBM treatment and still is the common mainstay therapeutic approach in the majority of patients [2]. The inability of conventional treatment to achieve durable remissions makes GBM an incurable disease [3, 4].

The heterogeneous nature of GBM results not only from genomic dissimilarities among different patients (intertumoral heterogeneity) but also from the plethora of cells composing the same specimen and showing different molecular characteristics and capacities to proliferate, migrate, invade [5] and sensitivity to treatment [6-8] (intratumoral heterogeneity). Likewise, GBM harbor a relative small population of cells showing stem properties [9, 10] defined as Glioblastoma-Initiating Cells (GICs), which are key contributors to treatment failure and poor patients outcome [11]. Several markers have been defined to isolate GICs from the bulk of the tumor: CD133, encoded by *PROM1* [12], CD44 [13], L1CAM [14] or ITGA6 [15]. Among them, CD133 is the most widely used, although a clear consensus on a unique marker or a combination of them to unequivocally identify GICs is still not available [16]. CD133^+^ GICs have been associated with radioresistance due to preferential activation of DNA-damage-response pathways [17, 18]. However, the over-reliance on CD133 as a unique marker of GICs led to contradictory results regarding stem properties [19, 20] and sensitivity to radiotherapy [21, 22] of CD133^-^ cells. Finally, transcriptomic analysis revealed four different GBM subtypes: Proneural (PN), Neural, Classical and Mesenchymal (Mes) [23]. Among them, tumors with a Mes gene signature are predominantly primary and, in some studies, show a more aggressive trend and greater resistance to radiation therapy than PN [24-26].

The purpose of this study was to address the relation between GICs and cell radioresponse, irrespective of any GICs marker expression. We established an *in-vitro* model with paired unsorted GICs and non-stem Differentiated Glioblastoma Cells (DGC) obtained from the same patient. Our established GICs displayed a clear Mes signature and when compared to DGCs counterpart, GICs were found to be more radioresistant to fractionated RT. In addition, we identified a direct correlation between *in-vitro* GICs radioresistance and patient outcome. Finally, we observed preferential upregulation of Mes markers and inflammation pathways in GICs acquiring a radioresistant phenotype. Our work contributes to improved understanding of the mechanisms involved in GBM radioresistance and may help point the way to the development of new therapeutic strategies for GBM patients.

## RESULTS

### Establishment of an *in-vitro* model of intratumoral GBM heterogeneity

**Figure 1:**
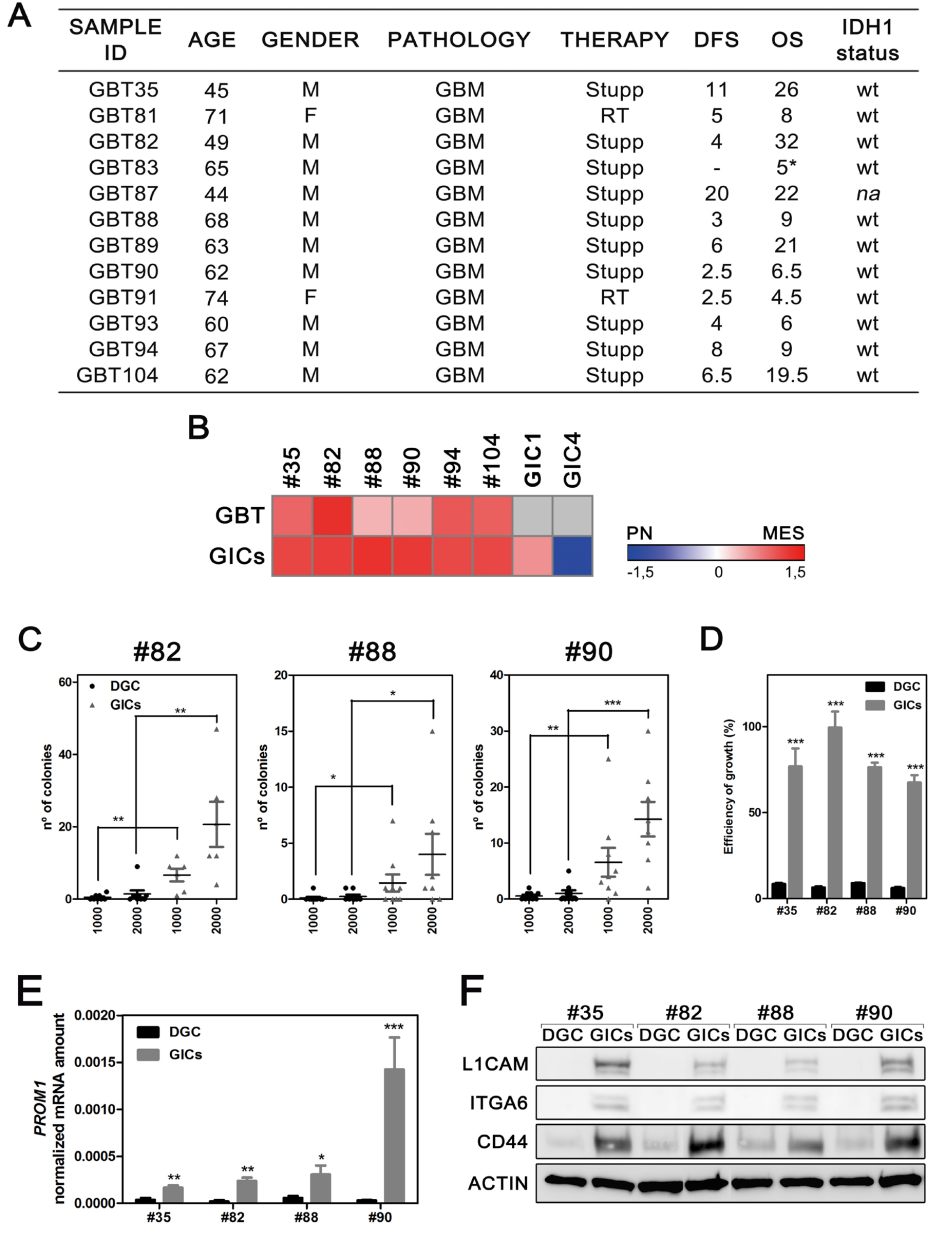
Establishment and characterization of the *in-vitro* model **A.** Clinical data of patients. M, male; F, female; DFS, disease-free survival; OS, overall survival; *na*, not analyzed (sample not available). * sudden death not related with GBM. **B.** Comparative heatmap of the molecular signature. Metagene score was calculated for each sample and compared to others after Z-score correction. Grey shade: undetermined data. **C.** Soft Agar assay performed in both culture conditions. The number of colonies scored was plotted respectively to the number of cell seeded (*n* = 9, *P < 0.05, **P < 0.01, ***P < 0.001, Mann-Whitney test). **D.** Plating efficiency of DGC and GICs cultures. Data are plotted as mean growth ratio for either colonies or neurospheres in reference to cells seeded (*n* = 4, ***P < 0.001, Mann-Whitney test). **E.** Real-time PCR analysis of the GICs marker *PROM1* (*n* = 4, *P < 0.05, **P < 0.01, ***P < 0.001, unpaired *t*-test). **F.** Western blot analysis of CD44, ITGA6 and L1CAM GICs markers.

GBM cell heterogeneity results from a wide-range of features including differences between DGC within the tumor bulk and GICs [27]. To analyze differences between these two cellular compartments and evaluate their response to radiotherapy, we generated an *in-vitro* model from 12 newly diagnosed GBM IDH1-wt tumors (GBT) (Figure 1A). Tumor cells from each patient were grown as both neurospheres and FBS-differentiated cultures to enrich for GICs and DGC, respectively. Six GICs/DGC culture pairs were successfully established and maintained *in-vitro* (GBT35, GBT82, GBT88, GBT90, GBT94 and GBT104).

We assessed whether the molecular profile of primary tumors was conserved during cell culture analyzing both, original tumor tissues and neurosphere-derived cultures, according to molecular subtype classification (Mesenchymal, *Mes*; and Proneural, PN). Using a set of four PN (*SOX9*, *OLIG2*, *SOX2* and *PROM1*) and four *Mes* (F*N1*, *CHI3L1*, *CD44* and *CTGF*) genes, we calculated the metagene score of each sample. All GBT exhibited a *Mes* score, which was maintained in neurosphere cultures (Figure 1B and Supplementary Figure S1A). Positive controls, with strong PN and *Mes* profiles, were included (Supplementary Figure S1B). Evaluation of MGMT promoter methylation status and major genomic alterations in primary tumor samples and the corresponding patient-derived culture pairs revealed consistent findings (Supplementary Figure S1C and S1D). Taken together, our results show that patient-derived cultures retained genetic characteristics of the original tumor.

### Cultured GICs express cancer stem cell (CSC) markers and display CSC functional features

To validate our neurosphere cultures as significantly enriched in GICs, we first analyzed their proliferative capacity by means of soft-agar assay and plating efficiency assay. GICs were more capable to proliferate at low cell density than their monolayer counterparts (Figure 1C, 1D and Supplementary Figure S2A). Primary neurosphere cultures also reported great capacity to self-renew independently of any paracrine stimuli and to undergo differentiation toward all CNS lineages (Supplementary Figure S2B and S2C). Finally, neurospheres were injected orthotopically in nude mice. By 7 weeks, histological analysis of xenografts revealed pathological features comparable to corresponding parental tumors (Supplementary Figure S2D).

We then evaluated a set of GIC markers in GICs/DGC culture pairs: CD133, CD44, L1CAM and ITGA6. GICs reported significant greater expression of *PROM1*, *L1CAM* and *ITGA6* at both mRNA and protein level, being selected markers almost undetectable in DGC (Figure 1E, 1F, Supplementary Figure S3A and S3B). Immunofluorescence staining was consistent with previous results (Supplementary Figure S3C). In addition, we identified within neurosphere cultures multiple clones expressing either L1CAM, ITGA6, CD44 or a combination of them, thus supporting GICs heterogeneity. Taken together, our results confirm that our primary neurosphere cultures were enriched in GICs and retained high level of heterogeneity.

### GICs intrinsic radioresistance is greater than DGC

**Figure 2:**
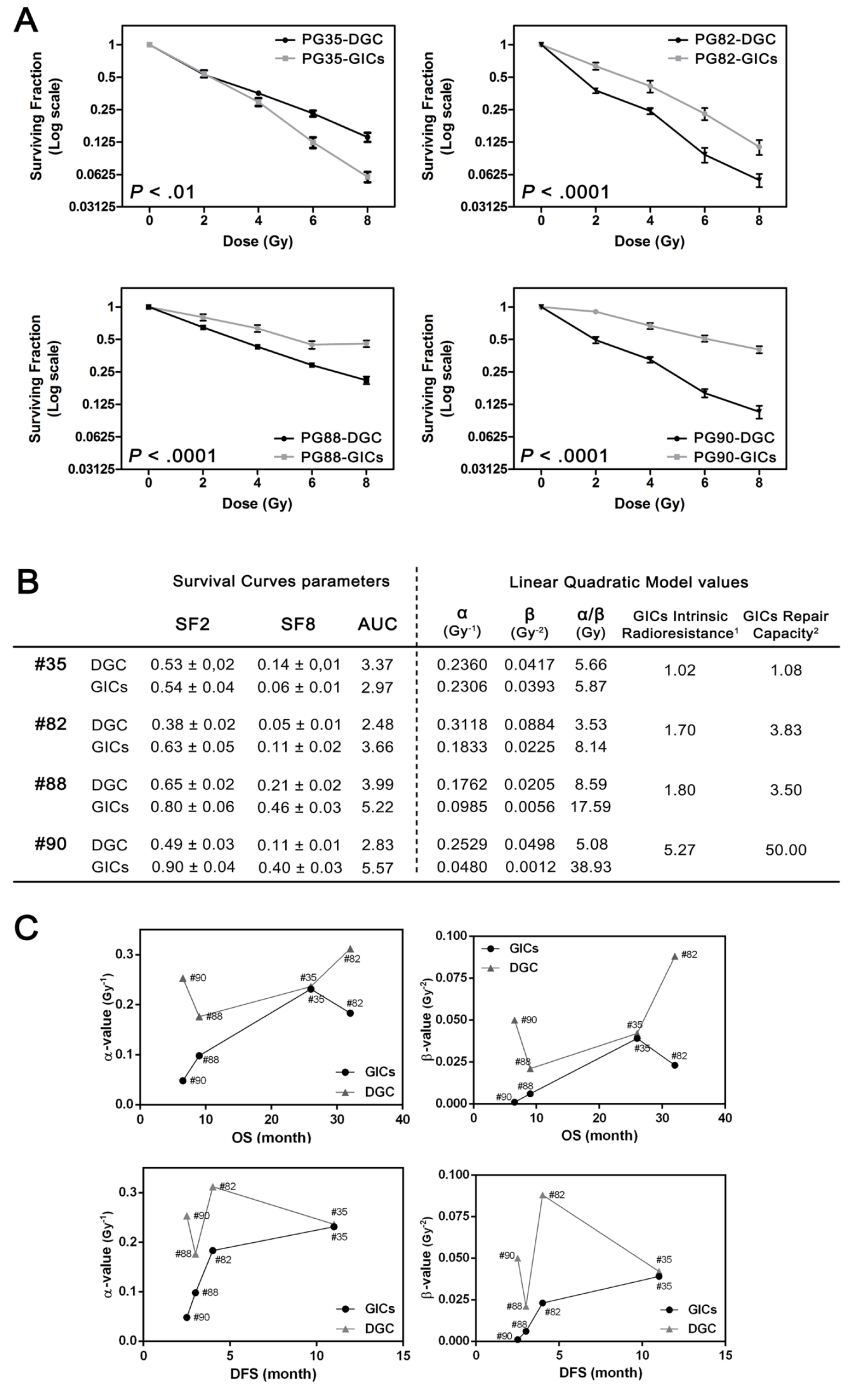
GICs exhibited higher radioresistance compared to DGC **A.** Survival curves after RT (*n* = 4, two-way ANOVA). Data are plotted in Log (2) scale. **B.** Survival curves and LQM parameters to quantify radiation sensitivity. SF2 and SF8 are indicated as mean ± SEM. ^(1)^ Calculated by the ratio α-DGC/α-GICs. ^(2)^ Calculated by the ratio β-DGC/β-GICs. SF2, surviving fraction at 2 Gy; SF8, surviving fraction at 8 Gy; AUC, area under the curve. **C.** Correlation plots among α- and β-values calculated for analyzed cultures and matching patient DFS and OS.

We next evaluated our patients-derived culture pairs responses to radiotherapy. We treated both DGC and GICs with fractionated RT at a dose of 2 Gy/day up to 8 Gy to simulate clinical radiotherapy regimens, and radiation response was evaluated by clonogenic assay (Figure 2A). Data of surviving curves were interpreted according to the Linear Quadratic Model (LQM) [28] by means of α- and β-values at 2 Gy. In terms of radiobiological meaning, α- and β- values correspond to intrinsic radiosensitivity and to repair capacity, respectively [29]. We detected different degrees of radiosensitivity among the evaluated GICs, being PG90-GICs the most radioresistant and PG35-GICs the least (Figure 2B). Interestingly, in three out of four culture pairs analyzed, GICs showed higher SF2, SF8 and AUC compared to DGC, suggesting a more radioresistant phenotype (#82, #88 and #90; *P* < 0.001). Likewise, the α- and β-parameters of GICs compared to their corresponding DGCs were markedly lower, indicating greater intrinsic radioresistance and capacity to repair radiation-induced DNA damage, respectively.

### Intrinsic radioresistance of GICs predicts clinical outcome

Next, we compared the LQM parameters obtained from each culture pair with the corresponding patient disease-free survival (DFS) and overall survival (OS). All patients were managed according to Stupp regimen [30]. Interestingly, we observed a significant correlation between patient’s DFS and its matching GICs α- and β-values (Bilateral Spearman’s rho *P* < 0.01): the smaller the α- and β-, the shorter the patient’s DFS (Figure 2C). In addition, patient GBT35, whose GICs culture displayed the highest α- and β-values, was the unique patient with complete response after concomitant RT and Temozolomide. Regarding OS, we also found a positive association with each patient α- and β-values. On the contrary, α- and β-parameters of the corresponding DGC compartment did not correlate with patient outcome. Taken together, these findings suggest that the overall clinical response to fractionated RT might rely more on the intrinsic radiosensitivity of their GICs subpopulation.

### Exposure of GICs to repeated cycles of RT promotes the acquisition of radioresistance

**Figure 3:**
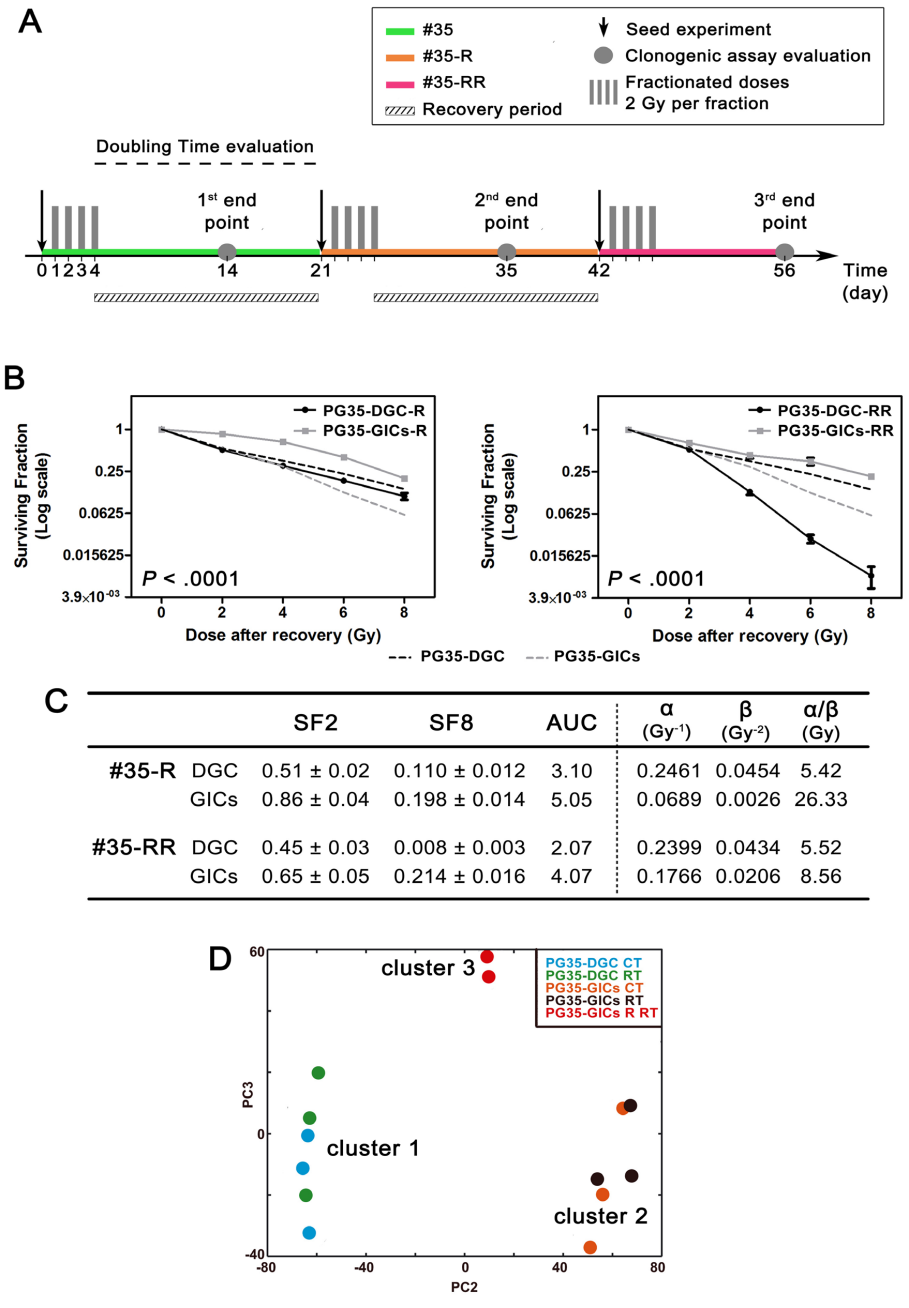
Generation of a radioresistant clone **A.** Schedule of the repeated cycles of fractioned RT carried out on #35 culture pair. **B.** Clonogenic assay survival curves after second (left) and third (right) radiation cycles. Data are plotted in Log(2) scale. Black and grey dashed lines represent PG35-DGC and PG35-GICs survival curves respectively and were included as internal reference (*n* = 4, two-way ANOVA calculated comparing PG35-DGC-R to PG35-GICs-R and PG35-DGC-RR to PG35-GICs-RR). **C.** Survival curves and LQM parameters. **D.** Genome-wide transcriptomic analysis of microarray data using unsupervised PCA plot. CT, control; RT, 8 Gy irradiated.

Then, we analyzed GICs responses to repeated cycles of RT. For these analyses, we chose the most radiosensitive culture pair established. PG35-DGC/GIC were exposed to standard 4-day cycle of fractionated doses scheduled every 3 weeks (Figure 3A). The recovery period within cycles was calculated according to complete restoration of the doubling time (data not shown). Following the first recovery period, cultures were named #35R where *R* stands for one completed cycle of radiation and recovery. Subsequently, PG35-DGC-R and PG35-GICs-R underwent a second cycle of radiation and recovery period (#35-RR). Finally, P#35-RR were treated with a third cycle of RT. Interestingly, the survival curve obtained from PG35-GICs-R revealed a significant switch toward a more radioresistant phenotype (Figure 3B). When compared to PG35-GICs, PG35-GICs-R displayed significantly higher SF2 and SF8 (*P* < 0.0001) with an overall statistical difference between the two curves (two-way ANOVA *P* < 0.0001; Figure 3C). Moreover, PG35-GICs-R curve showed an important reduction in α- and β-values at 2 Gy indicating increased intrinsic radioresistance and greater repair capacity, respectively. In contrast, PG35-DGC-R showed no statistical variation of SF2 or SF8 (*P* = 0.5 and *P* = 0.1, respectively). Comprehensively, PG35-GICs-R reported a significantly more radioresistant curve than PG35-DGC-R (*P* < 0.0001). Finally, after the third radiation cycle, PG35-DGC-RR reported dramatic radiosensitization (*P* < 0.0001) with a striking drop in SF8 (*P* < 0.0001), whereas PG35-GICs-RR maintained the acquired radioresistant phenotype (Figure 3B and 3C). In conclusion, repeated cycles of fractioned RT induced opposite responses in PG35-DGC and PG35-GICs based on the acquisition of different grades of radiosensitivity, which makes an attractive model for the study of determinants and regulators driving GICs acquired radioresistance.

### RT-induced radioresistant GICs predominantly express genes involved in inflammation, migration and EMT

Transcriptomes from fourteen samples representing five different #35 experimental conditions were analyzed using Human Gene 1.0 ST Array. PCA was conducted to visualize the overall transcriptome status of analyzed conditions (Figure 3D). Two major clusters reflecting culturing conditions but not irradiated status were identified: cluster_1 composed of PG35-DGC CT and RT samples, and cluster_2 composed of PG35-GICs CT and RT. Interestingly, irradiated PG35-GICs-R displayed a strong segregation from the above-mentioned clusters, thus defining a third cluster.

Comparative analysis of transcriptomes between CT and RT samples within the same cluster did not identify significant changes in gene expression (FDR < 0.05). However, GSEA identified some significant associations: after RT, DGC showed positive associations with inflammatory pathways and negative associations with several processes highlighting deregulation of cell-cycle and of chromosomal stability (Supplementary Figure S4). Single-treated GICs also displayed positive association with inflammatory pathways but unlike DGC, any pathway reported negative associations below FDR < 0.05 or nominal *p*-values < 0.05.

**Figure 4:**
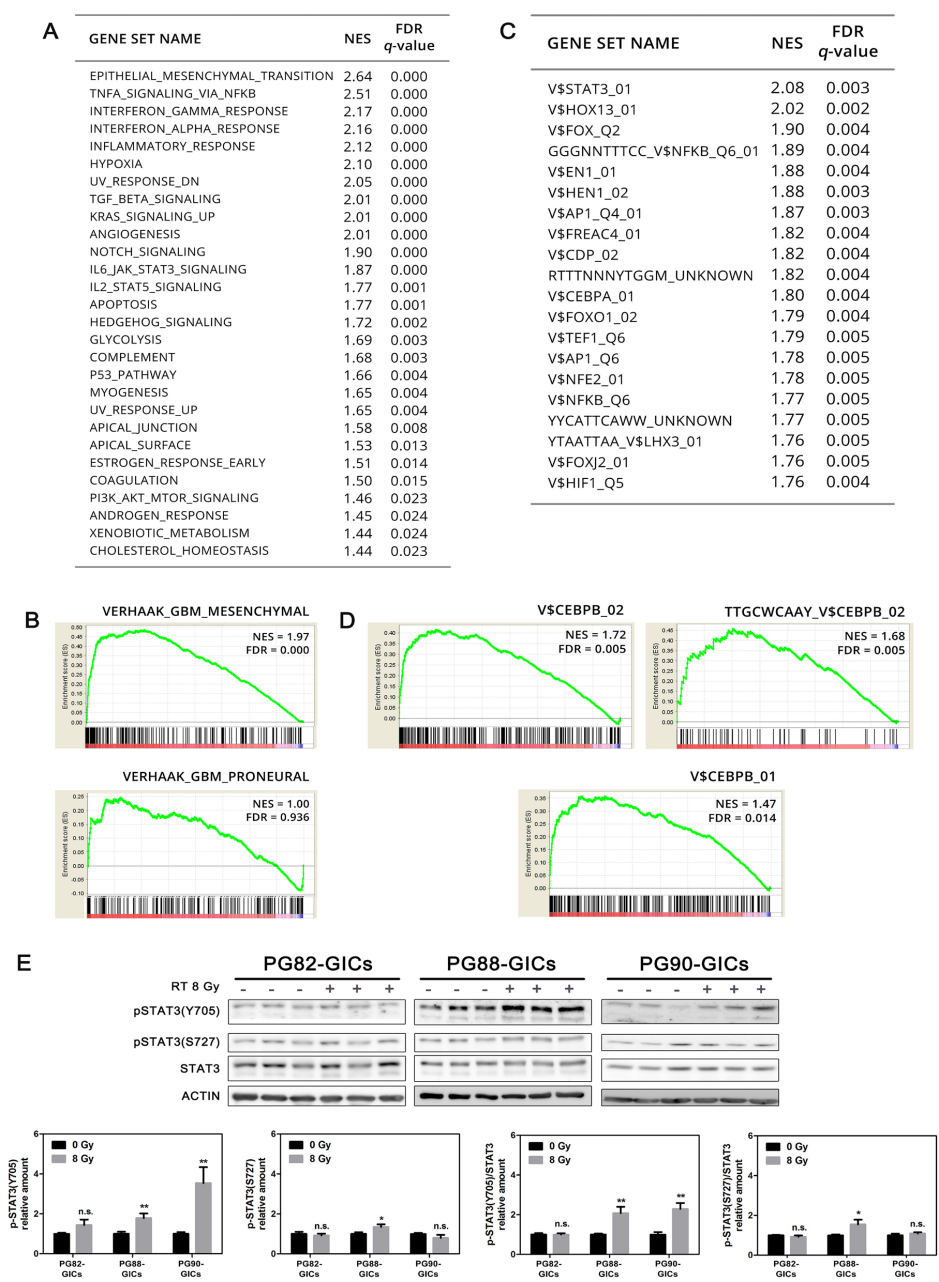
Transcriptomic analysis of radioresistant PG35-GICs-R **A.** List of GSEA Hallmark gene sets significantly enriched in cluster_3 compared to cluster_2 ranked according to NES. GSEA significance level: FDR < 0.05. NES, normalized enrichment score; FDR, false discovery rate. **B.** GSEA enrichment plot of Mes and PN Verhaak signatures [23]. **C.** Top twenty TF gene sets significantly activated in cluster_3 compared to cluster_2 ranked according to NES (FDR < 0.05). **D.** GSEA enrichment plot of C/EBPβ-associated gene sets. Individual NES and FDR were reported. **E.** Analysis of STAT3 phosphorylation following RT. Representative western blot and pSTAT3(Y705) and pSTAT3(S727) bands quantitation (*n* = 3, *P < 0.05; **P < 0.01, unpaired *t*-test).

To identify determinants of the radioresistant switch, irradiated PG35-GICs-R (cluster_3) was compared to cluster_2. GSEA-BioCarta identified several positively enriched pathways related to inflammation processes (Supplementary Figure S5), with *IL6*, *IL8* and *CSF3* among the genes with the highest scores (Supplementary Table S1). KEGG and Reactome databases highlighted enrichment of pathways regulating migration, Extracellular-Matrix (ECM) remodeling and cell-to-cell or cell-to-ECM interaction (Supplementary Figure S5). To gain more insight into the biological significance of our results, GSEA was interrogated through the Hallmark collection. Interestingly, genes associated with Epithelial-to-Mesenchymal Transition (EMT) and with the inflammatory response mediated by TNF-α, IFN-γ, IFN-α and IL-6 were the most enriched in double irradiated PG35-GICs (Figure 4A). To determine whether repeated radiation cycles induced a global shift in PG35-GICs toward a more Mes transcriptome, a GSEA on PN and Mes signatures obtained from TCGA network [23] was performed. Strikingly, cluster_3 compared to cluster_2 exhibited a highly significant enrichment of the mesenchymal signature (FDR < 0.001), but not of the PN one (FDR = 0.94; Figure 4B).

Finally, to identify which transcription factor (TF) might be involved in the observed transcriptional changes, a GSEA-TFT analysis was performed. Interestingly, the most significantly enriched TF in cluster_3 compared to cluster_2 were key TFs driving the PMT [24, 31], such as STAT3 , NF-κB and C/EBPβ (Figure 4C and 4D). To validate the activation of STAT3, phosphorylation in Tyr705 and Ser727 was evaluated. Interestingly, upon RT PG88-GICs and PG90-GICs (the more radioresistant cultures) showed a significant phosphorylation increase at Y705, but not at S727 residues, recently correlated with STAT3 mitochondrial functions [32] (Figure 4E).

### GICs upregulate a defined set of genes with prognostic value upon radiation

**Figure 5:**
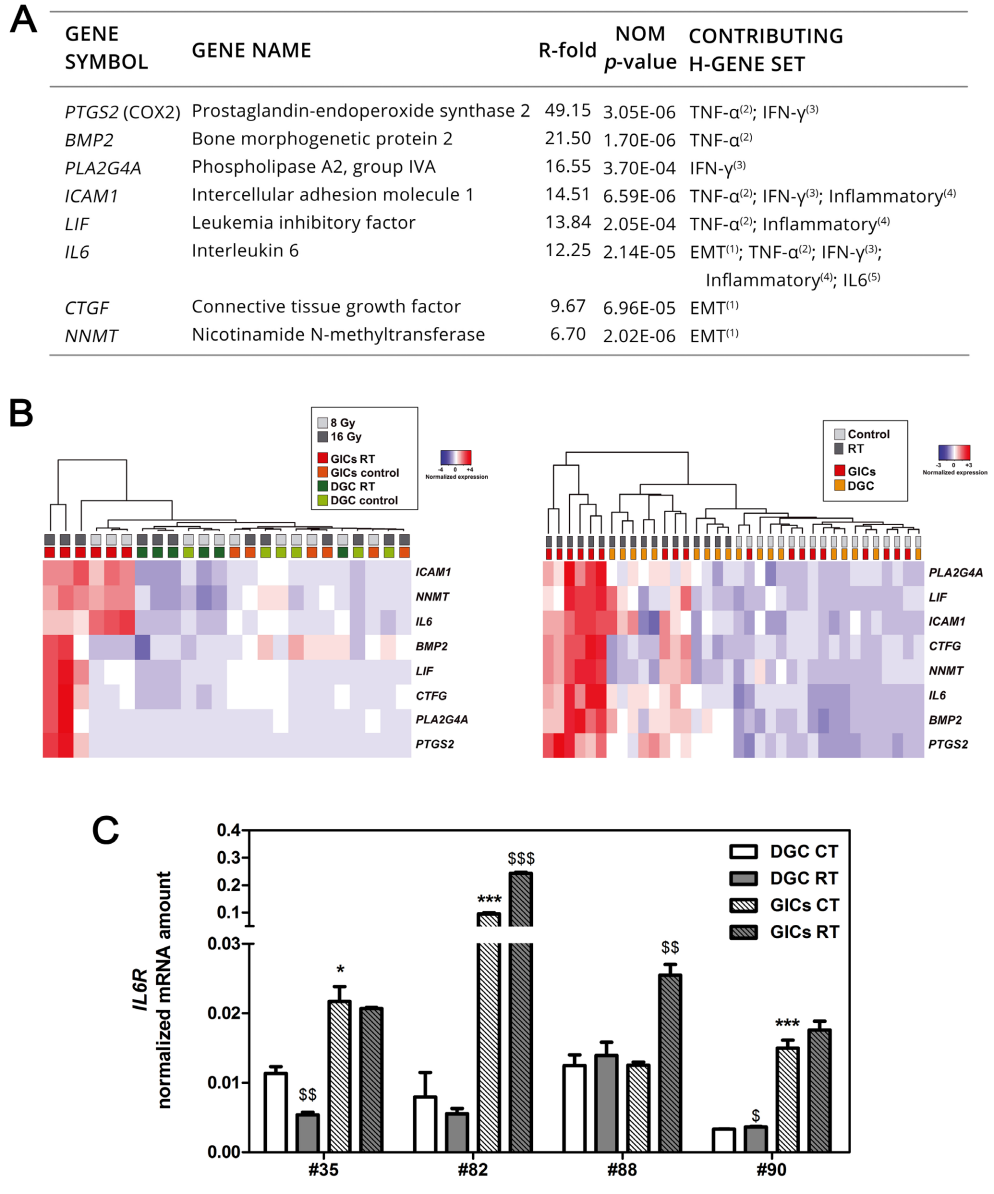
PMT and inflammation are preferentially activated in GICs following RT and correlate with patient outcomes **A.** Panel of genes selected to validate microarray data ranked according to R-fold (FDR < 0.05). **B.** Unsupervised hierarchical clustering of panel expression following fractionated RT and control condition. Data from #35 (left) and #82, #88, #90 (right) are depicted. **C.** IL6Rα expression in primary cultures (*n* = 3, *P < 0.05; ***P < 0.001, unpaired *t*-test comparing data to DGC CT; ^$$^*P* < 0.01; ^$$$^*P* < 0.001, unpaired *t*-test comparing data to corresponding control).

To verify the reliability of microarray findings, a panel was compiled based on the most upregulated genes with FDR < 0.05 and on their contribution to PMT and inflammatory pathways (Figure 5A). The induction of these genes was confirmed by q-PCR in #35 culture pair immediately after fractionated 8 Gy and 16 Gy (#35-R). After the second cycle of RT the whole genes increased their expression in GICs but not in DGC (Figure 5B). Other analyzed culture pairs reported comparable findings (Figure 5B). Next, to further explore the IL6/STAT3 pathway, we analyzed the expression of IL6Rα. Consistently with previous findings [33], all GICs reported either higher *IL6R* baseline expression or greater induction following RT (Figure 5C). We also found a good correlation between the defined panel with the GBM Mes subtype (Supplementary Figure S6A and S6B) and patient outcome (Supplementary Figure S6C). Taken together, these findings identify pathways, processes and specific genes related to mesenchymal GICs influencing GBM radiotherapy response and clinical outcome.

## DISCUSSION

Given the intratumoral heterogeneity and the increasing relevance of GICs in GBM recurrence [11], in this study we sought to develop an *in-vitro* model to investigate molecular mechanisms underlying GBM radioresistance based on these major cornerstones.

Despite the historical relevance of CD133 as a GIC marker [10], several studies have demonstrated that GBM cells lacking CD133 are unexpectedly capable of tumor initiation [19, 20]. Consequently, to avoid the underestimation of the stem pool and better preserve the heterogeneity of GICs, we chose the neurosphere culture method [9]. Established GICs clearly display mesenchymal traits, greater clonogenic capacity, have greater self-renewal and tumorigenic capability, and express more L1CAM, CD44, CD133 and ITGA6 compared to the non-GIC tumor bulk cells. Importantly, GICs display a heterogeneous compartment characterized by multiple clones.

Bao and colleagues reported higher radioresistance of CD133^+^ GICs when compared to CD133^-^ [17]. Yet, the simplified association between stemness and CD133 expression might have led to misinterpretations, since other studies analyzing CD133^+^ *versus* CD133^-^ found contradictory results [21, 22]. In our study, we demonstrate that all GIC-enriched cultures, established irrespectively of the expression of any stem cell marker, ended up being more radioresistant than their differentiated counterparts. Other groups investigated response to RT on similar paired culture systems, but the evaluation through cytometry-based analysis shortly after RT probably led to an underestimation of the RT long-term effects [34] and to claim a lack of significant difference between GICs and non-GICs cultures [35, 36]. Most importantly, we found that LQM parameters of GICs cultures positively correlated with patient outcome: the smaller the α- and β-values of GICs, the shorter the DFS of the matched patient. Although further studies with a wider cohort of paired samples is required, our findings support the key role of GICs in defining patient treatment response to RT.

The transcriptomic analysis of irradiated samples allowed us to identify molecular determinants associated with the acquisition of PG35-GICs-R radioresistance. Microarray analysis revealed a marked upregulation of pathways related to inflammation, PMT, ECM remodeling and cell migration. It is well known that beyond the induction of DNA damage, IR triggers complex inflammatory cascades in tumor and immune cells inducing the expression of interleukins (IL1, IL6, IL8, TNF-α) [37], adhesion molecules (ICAM-1, VCAM) [38] and activation of NF-kβ pathway, the central linker between inflammation, carcinogenesis and radioresistance [37, 39]. Apart from NF-kβ, STAT3 is a further inflammatory molecule activated by RT playing a crucial role in radioresistance of tumors [40-42]. The involvement of STAT3 in GBM radioresistance [43], lead us to analyze the STAT3 phosphorylation status following RT. Indeed, we detected RT-dependent activation of STAT3-pY705 in our established GICs. Moreover, IL6/JAK/STAT3 and TNF-α/NF-kβ pathways were significantly activated following RT, indicating and activation of inflammatory genes upon radiation. Thus, targeting the inflammatory signaling pathways induced by IR offers the opportunity to improve the clinical outcome of radiation therapy by enhancing radiosensitivity.

On the other hand, STAT3 and NF-κβ together with C/EBPβ act as master regulators of PMT [24, 31, 44]. In PG35-GICs-R these transcription factors and further PMT key pathways were significantly activated following RT. It is widely accepted that GBMs upon recurrence tend to shift from the PN toward the Mes subtype [45] and that RT can induce this transition in PN-GBM cells both *in-vitro* [46] and *in-vivo* [47]. Importantly, we show an enhancement of Mes signature upon RT also in our established Mes GICs, thus reinforcing the relevance of mesenchymal differentiation in GBM treatment resistance. Taken together, RT drives GICs toward an enriched Mes status independently of their original molecular subtype.

Following clinically relevant fractionated RT, our GICs but not DGC exhibited a significant upregulation of *ICAM1, PLA2G4A, PTGS2, LIF, IL6* and *NNMT* genes, indicating GICs preferential activation of inflammatory pathways and NNMT-mediated SSB repair [48]. In addition, we found a greater IL6Rα expression in GICs compartment suggesting a higher responsiveness to secreted IL6-LIF and an effective IL6-LIF/STAT3 autocrine loop. Moreover, we may speculate a juxtacrine effect of radiotherapy induced cytokines on stromal and cancer cells [49-51].Taken together, GICs secretome may drive a radiotherapy-induced shift toward a Mes status and the acquisition of a more aggressive phenotype.

We should mention, however, some limitations of the study. Although our results clearly suggested a correlation between *in-vitro* GICs radioresistance and patient outcome, the sample size is relatively small and hence a larger set of paired samples would be necessary to confirm our findings. In addition, further experiments should be carried out to address the involvement of inflammatory pathways in GBM radioresistance and to provide additional insights.

In summary, we propose an affordable *in-vitro* method as a tool to better understand the mechanism underlying GICs radioresistance and potentially predict patient response to RT based on empirical data. Our findings collectively support the leading role of GICs in defining patient treatment response and the relevance of targeting inflammatory and PMT signaling pathways in conjunction with radiation treatment.

## MATERIALS AND METHODS

### Primary glioblastoma cell cultures

Glioblastoma tumor samples (GBT) were collected during surgery from consenting patients according to the protocol approved by the Ethics Committee of Hospital Universitari de Bellvitge (HUB; histological diagnosis GBM WHO grade IV, IDH1-wt) [52]. The assessment of IDH1 status was carried out at the Pathology Department of the *HUB*. The presence of IDH1 point mutation at codon 132 (R132H) was evaluated through immunohistochemistry using a R132H mutation-specific antibody (clone H09; Optistain). GBT samples were processed within 1h and grown as DGC cultures [53], or as GICs following the neurosphere culture method [9, 54] (see also Supplementary Methods).

### Real-time q-PCR analyses

Total RNA was extracted from cells using TRIsure (Bioline). mRNA was treated with DNase I RNase-free (Thermo Scientific) and retro-transcribed with High Capacity cDNA Reverse Transcription Kit (Life Technologies). Gene expression analyses were performed using validated Taqman^®^ Gene Expression Assays (Applied Biosystems). Data analysis unless otherwise specified, was based on the ΔCt method with *GAPDH* and *GUSB* as housekeeping genes. Metagene score of molecular subtypes was calculated [24].

### Western blot

Proteins were extracted with 0.3% CHAPs buffer supplemented with protease inhibitors. Densitometric analysis was carried out using Multi-Gauge software (FujiFilm Corporation). Further details for the specific antibodies are given in Supplementary Methods.

### Soft agar assay

Soft agar assay was performed over a 0.5% agar layer, with single-cell suspension dissolved in 0.3% agar. Cells were maintained in culture for three weeks and then stained with MTT 0.5 mg/ml for six hours. The number of stained colonies was scored with ImageJ (NIH, USA).

### Clonogenic capacity

GICs were seeded in 96-well plates as single-cell suspension at low densities in triplicate. Cells were maintained for 14 days in their culturing media and then plates were visually scanned under light microscope. Neurospheres bigger than 100 µm were scored (ProgRes CapturePro). DGC were seeded in 6-well plates in triplicate and cells were stained after 14 days with 0.2% crystal violet and fixed with 2% ethanol. Colonies containing more than 50 cells were scored.

### Radiation schedule

Treatment of cells was carried out using an X-ray accelerator (Clinac 600 CD, M/S Varian AG) at a dose-rate of 2.67 Gy/min. Samples were irradiated every 24 hours following a fractionated schedule, using 2.0 Gy/fraction as repeated dose. Dosimetry calculations were performed by the Medical Physics Department at the Catalan Institute of Oncology.

### Clonogenic assay

To determine the radiation sensitivity of DGC cultures, clonogenic assay was performed as described [53]. For GICs cultures, the assay was adapted to the condition of free-floating spheres. GICs were seeded at low density (40 and 80 cells/well) in 96-well flat-bottomed plates and exposed to RT schedule 24 hours later. Following 14 days, the total number of newly formed neurospheres and colonies was recorded as indicated for clonogenic capacity assessment. Further details on the survival curve analysis are given in Supplementary Methods.

### Microarray analysis

A total of 100-300 ng of RNA was labelled using the WT Expression Kit (Ambion) and hybridized to Human Gene 1.0 ST Array (Affymetrix). Data were analyzed with Microarray Suite version 5.0 (MAS 5.0) using Affymetrix default analysis settings and global scaling as normalization method. The data were deposited under the GEO reference GSE82139 (http://www.ncbi.nlm.nih.gov/geo/query/acc.cgi?token = snclwmaerjytlyl&acc = GSE82139). The Gene Set Expression Analysis (GSEA) tool was run using default values for all parameters [55]. GSEA was interrogated using GSEA Hallmarks and transcription factor target (GSEA-TFT) enrichment and pathway annotation from Kyoto Encyclopedia of Genes and Genomes (KEGG), BioCarta (National Cancer Institute), and Reactome. Principal Component Analysis (PCA), unsupervised hierarchical clustering and heatmaps were obtained using the R (http://www.r-project.org/).

### Statistical analysis

Data graphs are presented as means ± SEM. Statistical analyses were performed using GraphPad Prism^®^ software.

## SUPPLEMENTARY MATERIALS FIGURES AND TABLE




