# Supplementary material for: Radioresistance of mesenchymal glioblastoma initiating cells correlates with patient outcome and is associated with activation of inflammatory program
Source: Oncotarget. 2017 Jun 3;8(43):73640–53. doi: 10.18632/oncotarget.18363 (PMC5650288; doi:10.18632/oncotarget.18363)
Supplement: Supplementary file 1 [file oncotarget-08-73640-s001.pdf]

Radioresistance of mesenchymal glioblastoma initiating cells correlates with patient outcome and is associated with activation of inflammatory program

SUPPLEMENTARY INFORMATION

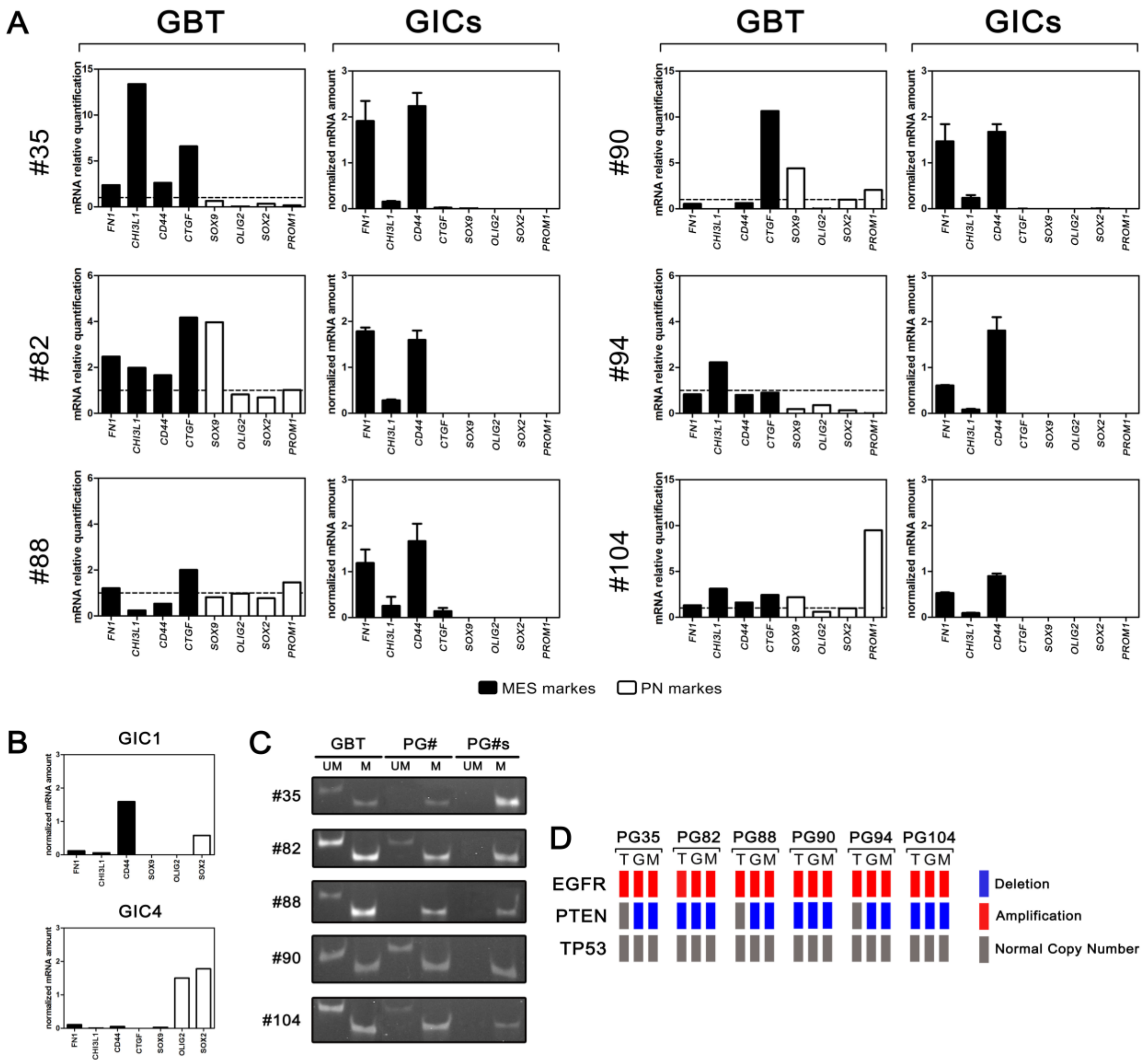

Supplementary Figure 1: Characterization of genomic and transcriptional features of established cultures

(A) GBM molecular subtype detection through qPCR analysis. Expression level of Mes (black bars) and PN (white bars) markers in parental tumors was calculated according to ΔΔCt method after normalization with *TBP* and *IPO8*. Each gene was then compared with its level in white matter of healthy donor brain (dashed line). Expression level in GICs cultures was depicted as normalized mRNA amount (n = 3). GBT, parental tumor.

(B) Expression level of Mes and PN markers in positive controls GIC1 and GIC4 displaying strong expression of Mes and PN trait, respectively.

(C) MGMT promoter methylation status of five sample trios. UM, unmethylated; M, methylated.

(D) MLPA for genomic copy number aberration of EGFR, PTEN and TP53. Ratios exclusively outside 0.8-1.2 range were accounted for as variations. Data shown are averages of all probes matching within each gene (n = 3). The major genomic alterations found in primary tumor tissues and the matching patient-derived culture pairs were consistent and included loss of PTEN locus and EGFR amplification. Of note, clear amplification of EGFR was detected in GBT82 and GBT104, whereas other samples require further validation. T, parental tumor; G, GICs cultures; D, DGC cultures.

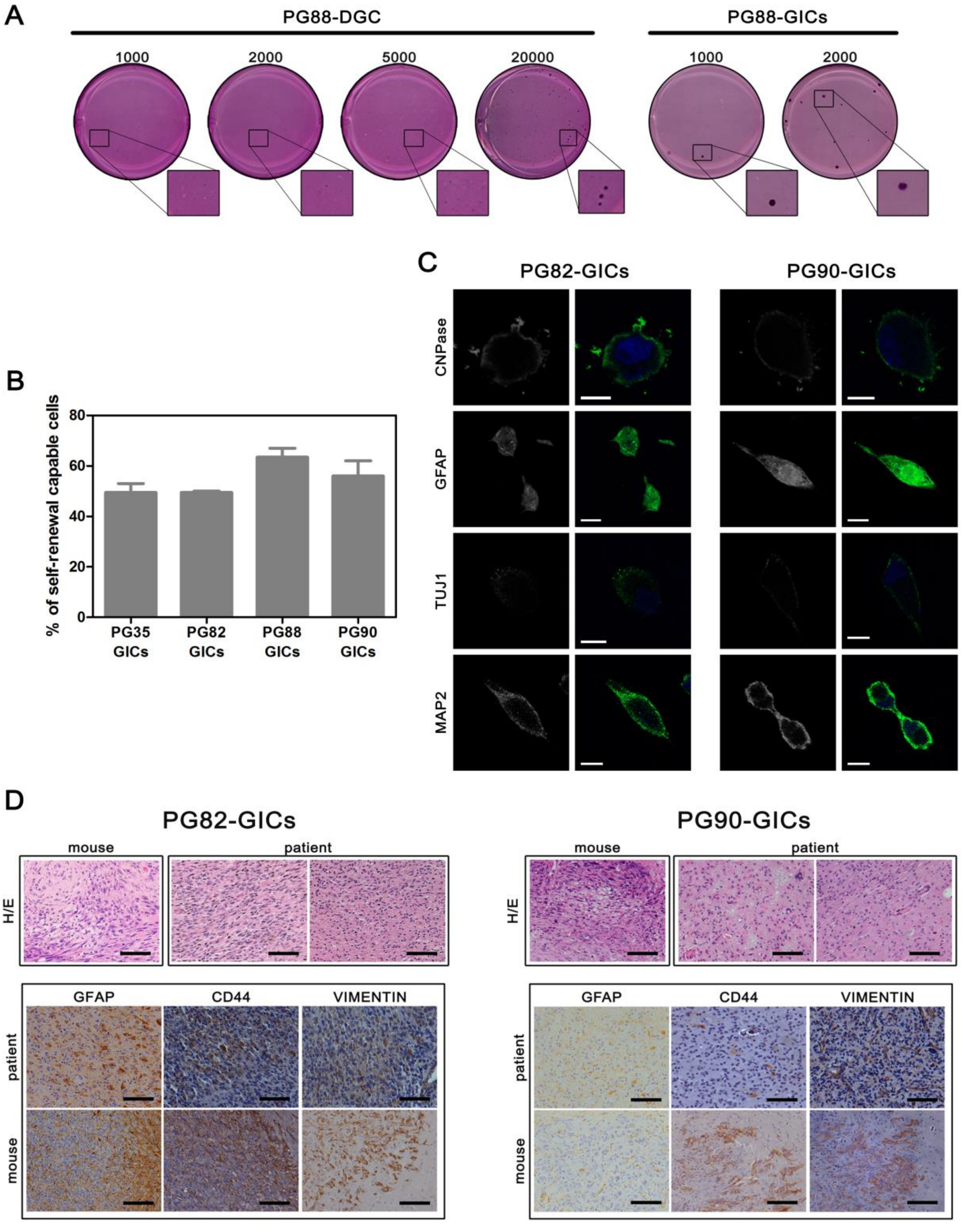

Supplementary Figure 2: Characterization of stem functional features of established GICs cultures

(A) Representative picture of Soft Agar Assay carried out with PG88-DGC and PG88-GICs. Cell seeded for each well is reported: 1000 and 2000 cell/well in PG88s; 1000, 2000, 5000 and 20000 cell/well in PG88. DGC cells had to be seeded 10-times more concentrated than neurospheres to appreciate real colony growth, and still the colonies generated by neurosphere cultures were considerably bigger.

(B) Self-renewal capability of neurosphere cultures. Data are plotted as percentage mean of cells capable of growing as spheres bigger than 100 µm in diameter after 14 days (n = 3 independent experiment).

(C) Immunofluorescence of differentiated PG82-GICs and PG90-GICs for neuronal markers (MAP2 and TUJ1), astrocytic marker (GFAP) and oligodendroglial marker (CNPase). Nuclei are counterstained with Draq5. Scale bar, 10 µm.

(D) Hematoxylin/eosin stain and immunohistochemistry for GFAP, CD44 and Vimentin. Tumor area generated in nude mouse brain following injection with PG82-GICs or PG90-GICs and the corresponding parental tumors were analyzed. Scale bar, 100 µm. Expression levels of the different proteins analyzed in mice and original tumors were found to be consistent.

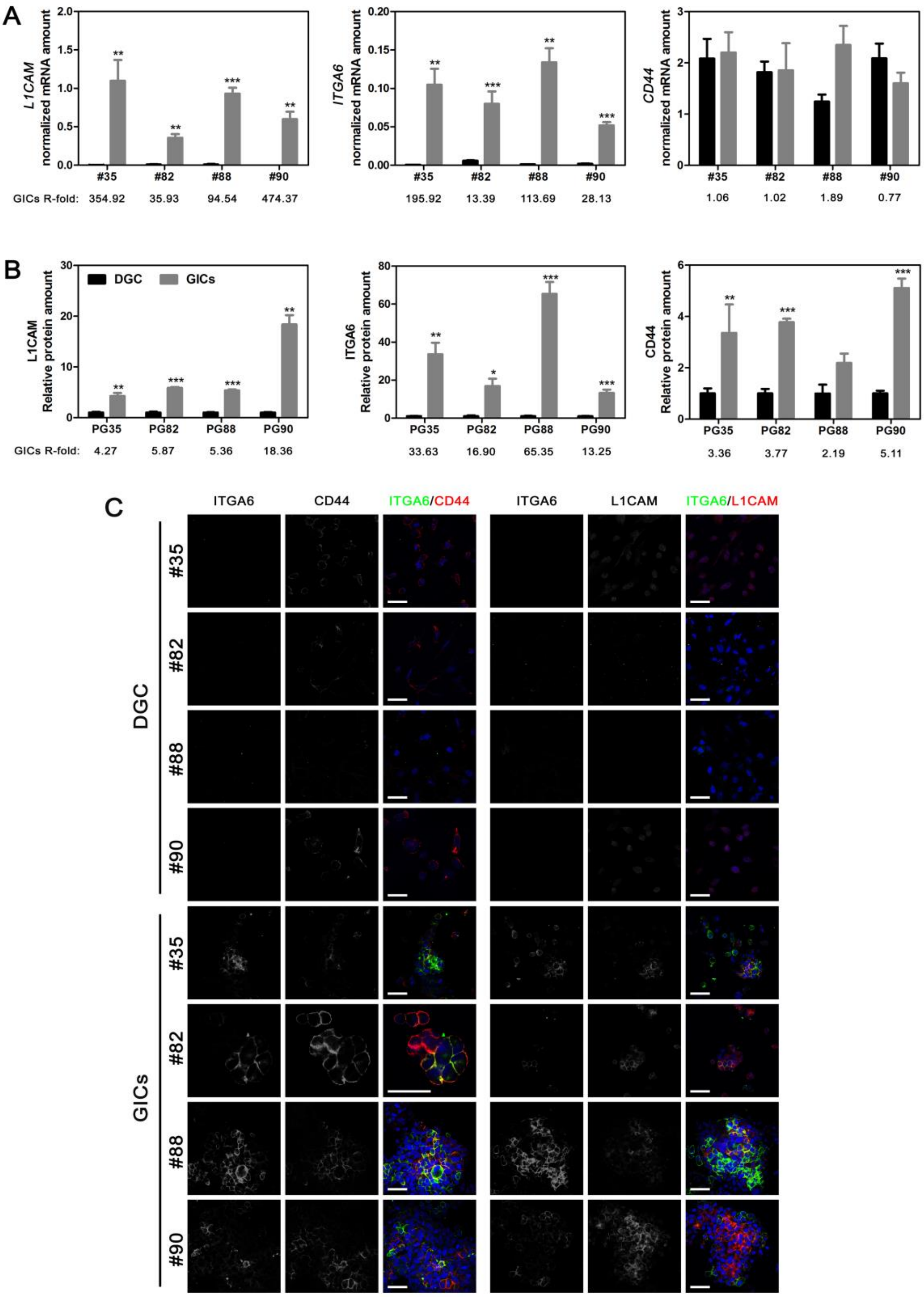

Supplementary Figure 3: Comparative detection of CSCs markers expression in established cultures

(A) Real-time PCR analysis of CSCs markers *L1CAM*, *ITGA6* and *CD44*. R-fold was obtained from data processed according to the ΔΔCt method, using corresponding DGC expression level as internal control (n = 3 with unpaired *t*-test, ** $P < .01$, *** $P < .001$).

(B) Western blot quantification of CD44, ITGA6 and L1CAM through densitometric analysis. Data are normalized to β-actin protein level (n = 3 independent samples with unpaired *t*-test, * $P < .05$, ** $P < .01$, *** $P < .001$). GICs R-fold indicates the fold difference between GICs culture and corresponding DGC. CD44 higher protein expression in GICs cultures was observed, suggesting protein stabilization.

(C) A representative picture of immunofluorescence analysis of selected GICs markers. Nuclei are counterstained with Draq5. Confocal images were taken with same settings between primary GICs and monolayer cultures in order to allow for effective comparison of fluorescence intensity. Scale bar, 50 µm.

DATABASE	GENE SET NAME	NES	FDR *q*-value	GENE SET BRIEF DESCRIPTION
Pathways positively enriched in PG35 after IR				
Reactome	GENERIC_TRANSCRIPTION_PATHWAY	2.53	0.000	*Genes involved in Generic Transcription Pathway*
KEGG	BLADDER_CANCER	2.03	0.000	*Alterated pathways in BC (RTK/Ras, p53/Rb, E-cad, MMPs and VEGF)*
BioCarta	LAIR_PATHWAY	1.97	0.038	*Cells and Molecules involved in local acute inflammatory response*
Reactome	NEGATIVE_REGULATORS_OF_RIG_I_MDA5_SIGNALING	1.95	0.039	*Genes involved in Negative regulators of RIG-I/MDA5 signaling*
KEGG	SMALL_CELL_LUNG_CANCER	1.90	0.016	*Molecular mechanisms altered in SCLC (MYC, p53, PTEN, RB, and FHIT)*
BioCarta	IL1R_PATHWAY	1.90	0.046	*Signal transduction through IL1R*
BioCarta	IL10_PATHWAY	1.87	0.044	*IL-10 Anti-inflammatory Signaling Pathway*
KEGG	EPITHELIAL_CELL_SIGNALING_IN_HELICOBACTER_PYLORI_INFECTION	1.80	0.040	*Epithelial cell signaling in Helicobacter pylori infection*
Pathways negatively enriched in PG35 after IR				
Reactome	AMYLOIDS	-3.03	0.000	*Genes involved in Amyloids*
Reactome	RNA_POL_I_PROMOTER_OPENING	-3.03	0.000	*Genes involved in RNA Polymerase I Promoter Opening*
Reactome	MEIOTIC_RECOMBINATION	-2.96	0.000	*Genes involved in Meiotic Recombination*
Reactome	RNA_POL_I_TRANSCRIPTION	-2.89	0.000	*Genes involved in RNA Polymerase I Transcription*
Reactome	MEIOSIS	-2.87	0.000	*Genes involved in Meiosis*
Reactome	DEPOSITION_OF_NEW_CENPA_CONTAINING_NUCLEOSOMES_AT_THE_CENTROMERE	-2.77	0.000	*Genes involved in Deposition of New CENPA-containing Nucleosomes at the Centromere (late telophase/early G1)*
Reactome	RNA_POL_I_RNA_POL_III_AND_MITOCHONDRIAL_TRANSCRIPTION	-2.76	0.000	*Genes involved in RNA Polymerase I, RNA Polymerase III, and Mitochondrial Transcription*
Reactome	CHROMOSOME_MAINTENANCE	-2.69	0.000	*Genes involved in Chromosome Maintenance (Telomeres maintenance and nucleosome assembly included)*
Reactome	MEIOTIC_SYNAPSIS	-2.67	0.000	*Stable physical pairing of homologous chromosomes*
Reactome	PACKAGING_OF_TELOMERE_ENDS	-2.62	0.000	*Processing and maintainance the Telomeres*
Reactome	TELOMERE_MAINTENANCE	-2.60	0.000	*Genes involved in Telomere Maintenance*
Reactome	MITOTIC_PROMETAPHASE	-2.48	0.000	*Genes involved in Mitotic Prometaphase (dissolution of nuclear membrane, Kinetochores creation, Microtubules attachment)*
Reactome	TRANSCRIPTION	-2.35	0.000	*Genes involved in Transcription*
Reactome	REGULATION_OF_BETA_CELL_DEVELOPMENT	-2.15	0.000	*Genes involved in Regulation of β-cell development*
Reactome	CELL_CYCLE	-2.05	0.002	*Genes involved in Cell Cycle regulation*
Reactome	REGULATION_OF_GENE_EXPRESSION_IN_BETA_CELLS	-1.99	0.004	*Genes involved in Regulation of gene expression in β-cells*
Reactome	KINESINS	-1.93	0.006	*Genes involved in Kinesins (motor proteins)*
Reactome	MITOTIC_M_M_G1_PHASES	-1.92	0.007	*Genes involved in Mitotic M-M/G1 phases*
Reactome	DNA_REPLICATION	-1.88	0.009	*Genes involved in DNA Replication*
Reactome	CHYLOMICRON_MEDIATED_LIPID_TRANSPORT	-1.79	0.021	*Genes involved in Chylomicron-mediated lipid transport*
Reactome	MUSCLE_CONTRACTION	-1.76	0.028	*Genes involved in Muscle contraction*
Reactome	DNA_STRAND_ELONGATION	-1.74	0.030	*DNA strand elongation at the DNA replication fork*
Reactome	RESOLUTION_OF_AP_SITES_VIA_THE_MULTIPLE_NUCLEOTIDE_PATCH_REPLACEMENT_PATHWAY	-1.70	0.039	*Genes involved in an alternative pathway of Base Excision Repair (BER)*
Reactome	EXTENSION_OF_TELOMERES	-1.68	0.045	*Genes involved in Telomeres Extension*
Pathways positively enriched in PG35s after IR				
Reactome	GENERIC_TRANSCRIPTION_PATHWAY	2.36	0.000	*Genes involved in Generic Transcription Pathway*
BioCarta	IL6_PATHWAY	2.10	0.005	*IL 6 signaling pathway*
KEGG	SPLICEOSOME	1.94	0.030	*Spliceosome*
Biocarta	TEL_PATHWAY	1.94	0.045	*Telomeres, Telomerase, Cellular Aging, and Immortality*

Supplementary Figure 4: GSEA gene sets positively and negatively enriched after the first IR cycle

List of gene sets significantly enriched after IR in PG35-DGC and PG35-GICs reported following GSEA nomenclature (FDR< .05). Gene sets were obtained by interrogating three different databases (Reactome, KEGG and BioCarta). FDR, false discovery rate; NES, normalized enrichment score.

GENE SET NAME	GENE SET BRIEF DESCRIPTION	NES	FDR *q*-value
**BioCarta**			
STEM_PATHWAY	*Regulation of hematopoiesis by cytokines*	2.21	0.001
EDG1_PATHWAY	*Phospholipids as signalling intermediaries*	2.16	0.001
INFLAM_PATHWAY	*Cytokines and Inflammatory Response*	2.15	0.001
IL1R_PATHWAY	*Signal transduction through IL1R (IL1 pro-inflammatory cytokine)*	2.13	0.001
LAIR_PATHWAY	*Cells and Molecules involved in local acute inflammatory response*	2.12	0.001
ERYTH_PATHWAY	*Erythrocyte Differentiation Pathway*	2.11	0.002
IL17_PATHWAY	*IL 17 Signaling Pathway (Inflammatory response)*	2.00	0.006
IL10_PATHWAY	*IL-10 Anti-inflammatory Signaling Pathway*	1.91	0.018
GSK3_PATHWAY	*Inactivation of Gsk3 by AKT*	1.89	0.022
NTHI_PATHWAY	*NFkB activation by Nontypeable Hemophilus influenzae*	1.86	0.027
SPPA_PATHWAY	*Aspirin Blocks Signaling Pathway Involved in Platelet Activation*	1.85	0.028
GPCR_PATHWAY	*Signaling Pathway from G-Protein Families*	1.84	0.031
P38MAPK_PATHWAY	*p38/MAPK Signaling Pathway*	1.83	0.030
MAPK_PATHWAY	*MAPKinase Signaling Pathway*	1.83	0.028
AT1R_PATHWAY	*Angiotensin II mediated activation of JNK Pathway via Pyk2 dependent signaling*	1.80	0.037
CARM_ER_PATHWAY	*CARM1 and Regulation of the Estrogen Receptor*	1.80	0.035
PPARA_PATHWAY	*Mechanism of Gene Regulation by Peroxisome Proliferators via PPARa(alpha)*	1.77	0.044
SHH_PATHWAY	*Sonic Hedgehog (Shh) Pathway*	1.77	0.042
VIP_PATHWAY	*Neuropeptides VIP and PACAP inhibit the apoptosis of activated T cells*	1.76	0.045
**KEGG**			
GLYCOSAMINOGLYCAN_BIOSYNTHESIS_KERATAN_SULFATE	*Glycosaminoglycan biosynthesis - keratan sulfate*	2.17	0.001
ECM_RECEPTOR_INTERACTION	*ECM-receptor interaction*	2.04	0.006
GLYCOSAMINOGLYCAN_BIOSYNTHESIS_CHONDROITIN_SULFATE	*Glycosaminoglycan biosynthesis - chondroitin sulfate*	2.00	0.006
HYPERTROPHIC_CARDIOMYOPATHY_HCM	*Hypertrophic cardiomyopathy (HCM)*	1.90	0.021
LEISHMANIA_INFECTION	*Leishmania infection*	1.87	0.023
SMALL_CELL_LUNG_CANCER	*Molecular mechanisms altered in SCLC (MYC, p53, PTEN, RB, and FHIT)*	1.78	0.049
PANCREATIC_CANCER	*Pathways activated in pancreatic cancer*	1.76	0.048
FOCAL_ADHESION	*Cell-matrix adhesions molecule (Integrin signaling)*	1.74	0.049
**Reactome**			
INTERFERON_ALPHA_BETA_SIGNALING	*Genes involved in Interferon alpha/beta signaling*	2.26	0.000
CHONDROITIN_SULFATE_DERMATAN_SULFATE_METABOLISM	*Chondroitin sulfate/dermatan sulfate metabolism*	2.13	0.003
A_TETRASACCHARIDE_LINKER_SEQUENCE_IS_REQUIRED_FOR_GAG_SYNTHESIS	*Genes involved in A tetrasaccharide linker sequence is required for GAG synthesis*	2.13	0.002
COLLAGEN_FORMATION	*Genes involved in Collagen formation*	2.05	0.009
INTEGRIN_CELL_SURFACE_INTERACTIONS	*Genes involved in Integrin cell surface interactions*	2.02	0.010
SIGNALING_BY_ROBO_RECEPTOR	*Genes involved in Signaling by Robo receptor*	2.02	0.009
GLYCOSAMINOGLYCAN_METABOLISM	*Genes involved in Glycosaminoglycan metabolism*	2.02	0.008
INTERFERON_GAMMA_SIGNALING	*Genes involved in Interferon gamma signaling*	2.01	0.008
KERATAN_SULFATE_KERATIN_METABOLISM	*Genes involved in Keratan sulfate biosynthesis*	1.98	0.012
MYOGENESIS	*Genes involved in Myogenesis*	1.97	0.012
KERATAN_SULFATE_BIOSYNTHESIS	*Genes involved in Keratan sulfate/keratin metabolism*	1.95	0.013
EXTRACELLULAR_MATRIX_ORGANIZATION	*Genes involved in Extracellular matrix organization*	1.95	0.012
INTERFERON_SIGNALING	*Genes involved in Interferon Signaling*	1.95	0.012
CIRCADIAN_CLOCK	*Genes involved in Circadian Clock*	1.91	0.019
RORA_ACTIVATES_CIRCADIAN_EXPRESSION	*Genes involved in RORA Activates Circadian Expression*	1.90	0.021
INSULIN_SYNTHESIS_AND_PROCESSING	*Genes involved in Insulin Synthesis and Processing*	1.84	0.039
ACTIVATION_OF_CHAPERONE_GENES_BY_XBP1S	*Genes involved in Activation of Chaperone Genes by XBP1(S)*	1.83	0.040
BMAL1_CLOCK_NPAS2_ACTIVATES_CIRCADIAN_EXPRESSION	*Genes involved in BMAL1:CLOCK/NPAS2 Activates Circadian Expression*	1.83	0.039
HEPARAN_SULFATE_HEPARIN_HS_GAG_METABOLISM	*Genes involved in Heparan sulfate/heparin (HS-GAG) metabolism*	1.83	0.038
SIGNALING_BY_PDGF	*Genes involved in Signaling by PDGF*	1.83	0.036
NRAGE_SIGNALS_DEATH_THROUGH_JNK	*Genes involved in NRAGE signals death through JNK*	1.81	0.043
CIRCADIAN_REPRESSION_OF_EXPRESSION_BY_REV_ERBA	*Genes involved in Circadian Repression of Expression by REV-ERBA*	1.80	0.045
HS_GAG_DEGRADATION	*Genes involved in HS-GAG degradation*	1.78	0.049

Supplementary Figure 5: GSEA gene sets positively enriched after the radioresistant switch

List of gene sets significantly enriched after double-IR in PG35-GICs-R reported following GSEA nomenclature (FDR< .05). Gene sets were obtained by interrogating three different databases (Reactome, KEGG and BioCarta). FDR, false discovery rate; NES, normalized enrichment score.

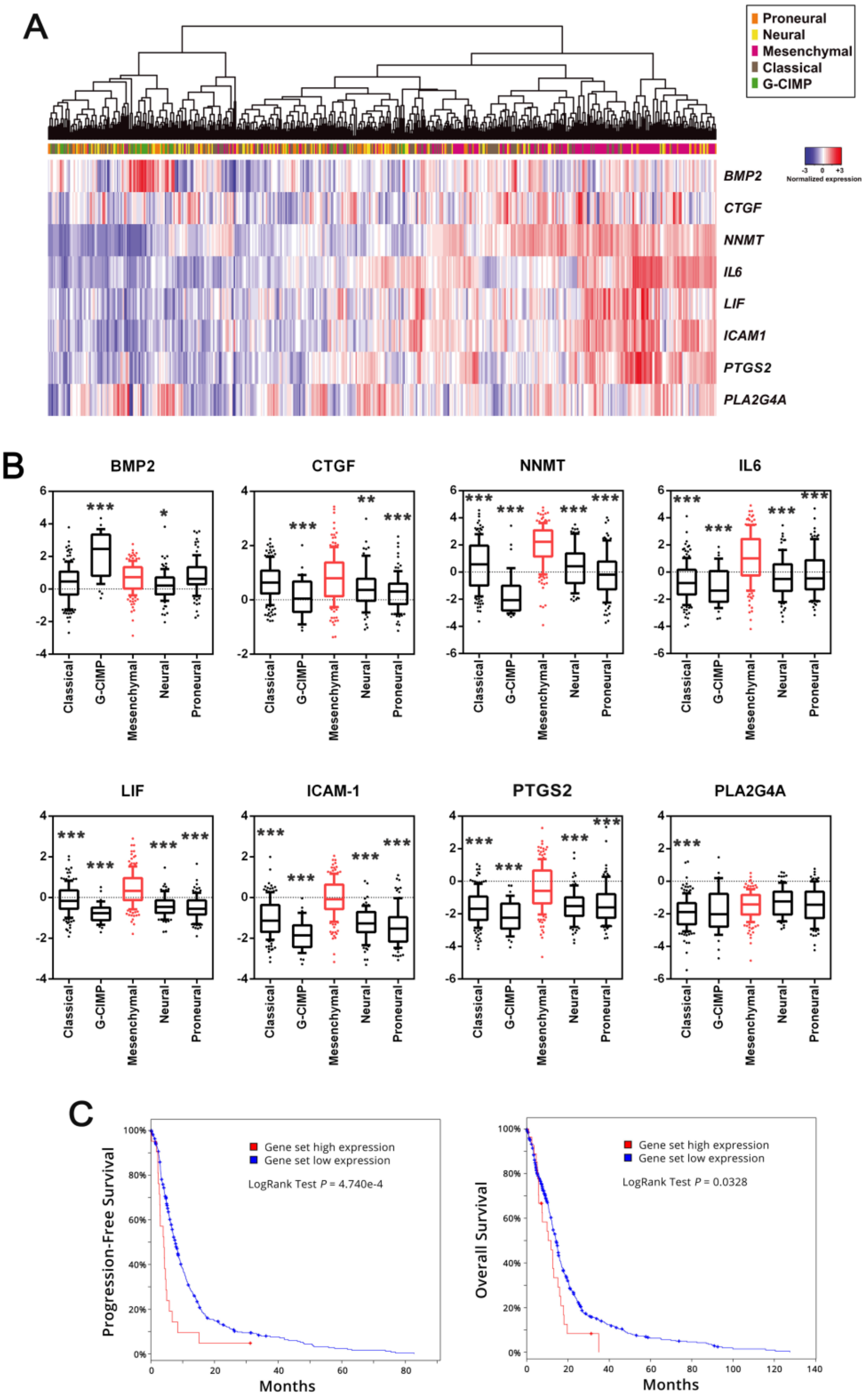

Supplementary Figure 6: Expression of most of the genes composing the selected panel showed significant upregulation in GBM Mes subtype

(A) Unsupervised hierarchical clustering of TCGA GBM samples (AgilentG4502A_07_2) according to expression of the selected panel. A significant association was detected between the panel expression and the GBM Mes subtype.

(B) Box-plot diagram showing the expression of the indicated genes across the different molecular subtypes of GBM. Data were obtained from the TCGA array platform (AgilentG4502A_07_2) extracted from the TCGA cancer browser (* $P < .05$, ** $P < .01$, *** $P < .001$ with ANOVA in comparison to expression in Mes subtype). Among gene composing the panel *PTGS2*, *LIF*, *ICAM1*, *IL6*, *CTGF* and *NNMT* reported a significantly higher expression in Mes subtype.

(C) Kaplan-Meier curves showing progression-free survival (left) and overall survival (right) of GBM patients with either no expression alteration or upregulation of *PTGS2, LIF, ICAM1, IL6, CTGF* and *NNMT*. According to cBioPortal default settings, median PFS was 4.07 months for patients with high expression of the gene set, whereas it was 7.62 months for those with low expression ($P < .001$). In addition, median OS was 11.83 and 14.62 months for patients with high and low expression, respectively ($P < .05$).

Table S1. List of genes differentially expressed in cluster 3 versus cluster 2

Genes significantly upregulated or downregulated following double-IR in PG35-GICs-R showing FDR< .05. Indicated nominal *p*-value was calculated with two-tailed unpaired *t*-test.

Supplementary Methods

Isolation of GICs from parental tumors

Samples were first rinsed with Hank's Balanced Salt Solution (HBSS), then mechanically dissociated and enzymatically digested with 20 U/ml Papain (Worthington) stabilized with 8.25 μM L-Cysteine (Sigma) and 3.42 μM EDTA (Panreac) for 30 min at 37ºC with constant agitation, and then cultured in FBS-free media [1]. Neurosphere primary cell cultures were subsequently split 1:10 every 5-7 days by mechanical dissociation and extensively maintained for at least twenty passages. All cultures were maintained at 37°C in a humidified atmosphere with 5% carbon dioxide and were tested for mycoplasma routinely. All experiments were performed before passage 20.

Western blot antibodies employed

The following antibodies were used: anti-L1CAM (1:1000; clone 2C2; Abcam), anti-ITGA6 (1:500; HPA012626; Novus Biologicals), anti-CD44 (1:100; clone 156-3C11; Thermo scientific), anti-β-Actin (1:5000; clone AC-15; Sigma), anti-pSTAT3-Y705 (1:2000; clone D3A7; CST), anti-pSTAT3-S727 (1:500; #78835; RD System) and STAT3 (1:2000; clone 79D7; CST). To avoid false negative signal in monolayer cultures during protein analysis, cells were collected by scraping, thereby avoiding trypsin digestion.

Interpretation of survival curves with the LQM

Surviving fraction (SF) of each treatment dose (D) was obtained according to previously described protocol [2]; SF data were then fit according to the linear–quadratic model (LQM): $SF = \exp -(\alpha D+\beta D^2)$. Differential radiosensitivity was evaluated by means of the surviving fraction after 2 Gy (SF2) and 8 Gy (SF8); the area under the curve (AUC) and the LQM parameters: α- and β-values and α/β [2–4].

Methylation-specific PCR

Genomic DNA was isolated from frozen tumor using the Qiamp DNA mini kit (Quiagen). DNA methylation status of CpG islands of the enzyme O6-methylguanine methyltransferase (MGMT) promoter was determined by methylation-specific PCR (MSP) as previously described [5].

Aberrant genomic copy number detection

Copy numbers of EGFR, TP53 and PTEN were detected through Multiplex Ligation-dependent Probe Amplification (SALSA MLPA Kit P105, MLPA®, Mrc-Holland, The Netherlands). Analysis was carried out on patient tumor tissues (GBT), and primary DGC and GICs cultures. DNA was extracted with Qiamp DNA mini kit (Quiagen), and MLPA was executed following the manufacturer's instructions. MLPA profiles of primary cultures and tumor samples were compared to genomic DNA obtained from healthy donors. Peak areas were analyzed with Coffalyser software (MRC Holland, The Netherlands) considering as normal variation values those falling between 0.8 and 1.2. Ratios below 0.8 were considered as locus deletions. Ratios above 1.2 were considered as amplifications.

Self-renewal assay

For the self-renewal assay, neurospheres were mechanically dissociated and seeded at extremely low cell density (1 cell per well) in a 96-well flat-bottomed plate. After 24 hours, plates were visually scanned with inverted light microscope to select wells containing a single cell. Colony formation was recorded 14 days later and the percentage of growth as neurosphere was determined. Only neurospheres exceeding 100 µm in diameter were counted (ProgRes CapturePro).

Intracranial tumor assay

All mouse experiments were approved by and performed according to the guidelines of the IDIBELL Animal Care Committee in agreement with the European Union and national directives. $1x10^5$ GICs were inoculated approximately into the corpus striatum of the right brain hemisphere of 7-week-old male athymic mice (Harlan). After 7 weeks mice were euthanatized, and brains were collected, formalin fixed and paraffin embedded. Immunohistochemistry was performed on 4 µm slices using the following primary antibodies: anti-GFAP (clone 6F2; Dako), anti-vimentin (clone 3B4; Dako) and anti-CD44 (clone 156-3C11; Thermo). Samples were counterstained with hematoxylin. Original patient tumor samples underwent the same procedures.

Immunofluorescence staining

DGC and GICs cultures were fixed in 4% paraformaldehyde for 20 min. To perform the analysis of free-floating neurospheres, a variation of the protocol was carried out as previously described [6]. The following antibodies were used: anti-L1CAM (1:1000; clone 2C2; Abcam), anti-ITGA6 (1:100; NKI-GoH3; Millipore) and anti-CD44 (1:100; clone 156-3C11; Thermo scientific). Micrographs were captured with a Leica TCS-SL Spectral confocal microscope (Leica Microsystems, Germany). Images were assembled using Adobe Photoshop, with identical adjustments for contrast, brightness and color balance.

Differentiation assay

In order to assess GICs capacity to differentiate along the mayor CNS lineages, cells were seeded and maintained with 10% FBS for 14 days. Samples were then processed for immunofluorescence. The following antibodies were used: anti-MAP2 (1:100; Sigma), anti-CNPase (1:100; Sigma), anti-GFAP (1:50; Dako) and anti-Tuj1 (1:1000; Covance).

Supplementary references

1. Gritti A, Parati EA, Cova L, Frolichsthal P, Galli R, Wanke E, Faravelli L, Morassutti DJ, Roisen F, Nickel DD, Vescovi AL. Multipotential Stem Cells from the Adult Mouse and Self-Renew in Response to Basic Fibroblast. J Neurosci. 1996; 16:1091–100.

2. Franken NA, Rodermond HM, Stap J, Haveman J, van Bree C. Clonogenic assay of cells in vitro. Nat Protoc. 2006; 1:2315–19.

3. Franken NA, van Bree C, ten Cate R, van Oven CH, Haveman J. Importance of TP53 and RB in the repair of potentially lethal damage and induction of color junctions after exposure to ionizing radiation. Radiat Res. 2002; 158:707–14.

4. Brenner DJ. The linear-quadratic model is an appropriate methodology for determining isoeffective doses at large doses per fraction. Semin Radiat Oncol. 2008; 18:234–39.

5. Villalonga-Planells R, Coll-Mulet L, Martínez-Soler F, Castaño E, Acebes JJ, Giménez-Bonafé P, Gil J, Tortosa A. Activation of p53 by nutlin-3a induces apoptosis and cellular senescence in human glioblastoma multiforme. PLoS One. 2011; 6:e18588.

6. Sasaki R, Aoki S, Yamato M, Uchiyama H, Wada K, Ogiuchi H, Okano T, Ando T. A protocol for immunofluorescence staining of floating neurospheres. Neurosci Lett. 2010; 479:126–27.
