# Supplementary material for: Radioresistance of mesenchymal glioblastoma initiating cells correlates with patient outcome and is associated with activation of inflammatory program
Source: Oncotarget. 2017 Jun 3;8(43):73640–53. doi: 10.18632/oncotarget.18363 (PMC5650288; doi:10.18632/oncotarget.18363)
Supplement: Supplementary file 2 [file oncotarget-08-73640-s002.docx]

**Supplementary Table 1. List of genes differentially expressed in cluster 3 versus cluster 2**

| **Probe ID** | **Gene symbol** | **Gene title** | **R-fold** | **P-value** |
| --- | --- | --- | --- | --- |
| 8097692 | EDNRA | endothelin receptor type A | 50.11 | 4.52E-04 |
| 7922976 | PTGS2 | prostaglandin-endoperoxide synthase 2 (prostaglandin G/H synthase and cyclooxygenase) | 49.15 | 3.05E-06 |
| 7962375 | PRICKLE1 | prickle homolog 1 (Drosophila) | 32.88 | 7.70E-06 |
| 8031646 | LOC100288114 | hypothetical LOC100288114 | 32.03 | 1.95E-06 |
| 8031646 | LOC100128252 | hypothetical LOC100128252 | 32.03 | 1.95E-06 |
| 8040430 | VSNL1 | visinin-like 1 | 29.15 | 1.27E-06 |
| 8046086 | LASS6 | LAG1 homolog, ceramide synthase 6 | 28.95 | 8.71E-07 |
| 8045664 | LYPD6B | LY6/PLAUR domain containing 6B | 22.64 | 1.44E-06 |
| 8112274 | ELOVL7 | ELOVL family member 7, elongation of long chain fatty acids (yeast) | 22.01 | 2.06E-08 |
| 8060850 | BMP2 | bone morphogenetic protein 2 | 21.50 | 1.70E-06 |
| 7983910 | AQP9 | aquaporin 9 | 21.33 | 2.52E-06 |
| 8001800 | CDH11 | cadherin 11, type 2, OB-cadherin (osteoblast) | 19.80 | 1.97E-05 |
| 8122150 | EYA4 | eyes absent homolog 4 (Drosophila) | 18.24 | 1.23E-07 |
| 7908204 | HMCN1 | hemicentin 1 | 18.08 | 6.54E-07 |
| 8096301 | SPP1 | secreted phosphoprotein 1 | 17.83 | 7.42E-04 |
| 8067955 | CXADR | coxsackie virus and adenovirus receptor | 17.18 | 4.03E-05 |
| 8047738 | NRP2 | neuropilin 2 | 17.15 | 1.22E-05 |
| 8127563 | COL12A1 | collagen, type XII, alpha 1 | 16.66 | 6.69E-07 |
| 7908351 | PLA2G4A | phospholipase A2, group IVA (cytosolic, calcium-dependent) | 16.55 | 3.70E-04 |
| 8166511 | PDK3 | pyruvate dehydrogenase kinase, isozyme 3 | 16.16 | 3.23E-06 |
| 7927681 | BICC1 | bicaudal C homolog 1 (Drosophila) | 15.79 | 6.64E-07 |
| 8097991 | TDO2 | tryptophan 2,3-dioxygenase | 15.14 | 1.75E-05 |
| 7908161 | C1orf21 | chromosome 1 open reading frame 21 | 14.83 | 4.43E-06 |
| 8045674 | LYPD6 | LY6/PLAUR domain containing 6 | 14.62 | 3.09E-06 |
| 8140579 | CACNA2D1 | calcium channel, voltage-dependent, alpha 2/delta subunit 1 | 14.56 | 3.84E-06 |
| 8025601 | ICAM1 | intercellular adhesion molecule 1 | 14.51 | 6.59E-06 |
| 8160346 | PTPLAD2 | protein tyrosine phosphatase-like A domain containing 2 | 14.36 | 8.05E-05 |
| 8075310 | LIF | leukemia inhibitory factor (cholinergic differentiation factor) | 13.84 | 2.05E-04 |
| 8081838 | ARHGAP31 | Rho GTPase activating protein 31 | 13.60 | 3.51E-04 |
| 8040103 | ID2 | inhibitor of DNA binding 2, dominant negative helix-loop-helix protein | 13.56 | 1.88E-04 |
| 8152606 | SNTB1 | syntrophin, beta 1 (dystrophin-associated protein A1, 59kDa, basic component 1) | 13.31 | 2.02E-06 |
| 8126760 | RCAN2 | regulator of calcineurin 2 | 13.14 | 1.36E-04 |
| 7965403 | LUM | lumican | 12.83 | 5.46E-04 |
| 8113039 | MEF2C | myocyte enhancer factor 2C | 12.41 | 3.55E-05 |
| 8113433 | EFNA5 | ephrin-A5 | 12.36 | 7.96E-07 |
| 8131803 | IL6 | interleukin 6 (interferon, beta 2) | 12.25 | 2.14E-05 |
| 7939052 | FIBIN | fin bud initiation factor homolog (zebrafish) | 11.96 | 4.26E-07 |
| 8091402 | TM4SF18 | transmembrane 4 L six family member 18 | 11.76 | 5.02E-06 |
| 8044574 | IL1RN | interleukin 1 receptor antagonist | 11.56 | 5.28E-05 |
| 7903227 | PALMD | palmdelphin | 11.40 | 1.62E-07 |
| 8081686 | BOC | Boc homolog (mouse) | 11.22 | 5.32E-05 |
| 8023415 | TCF4 | transcription factor 4 | 10.88 | 2.21E-04 |
| 8082965 | MRAS | muscle RAS oncogene homolog | 10.66 | 1.32E-06 |
| 7972003 | KLF12 | Kruppel-like factor 12 | 10.65 | 2.81E-05 |
| 7902353 | LHX8 | LIM homeobox 8 | 10.49 | 7.50E-05 |
| 8180411 | ELMO1 | engulfment and cell motility 1 | 10.41 | 6.92E-07 |
| 8104592 | FBXL7 | F-box and leucine-rich repeat protein 7 | 10.34 | 8.96E-07 |
| 8148435 | WISP1 | WNT1 inducible signaling pathway protein 1 | 10.15 | 5.76E-07 |
| 7985159 | CRABP1 | cellular retinoic acid binding protein 1 | 10.09 | 5.35E-06 |
| 8078330 | RBMS3 | RNA binding motif, single stranded interacting protein 3 | 10.06 | 3.09E-05 |
| 8120961 | MRAP2 | melanocortin 2 receptor accessory protein 2 | 9.72 | 4.62E-05 |
| 8144726 | TUSC3 | tumor suppressor candidate 3 | 9.67 | 2.90E-06 |
| 8129562 | CTGF | connective tissue growth factor | 9.67 | 6.96E-05 |
| 8066848 | PREX1 | phosphatidylinositol-3,4,5-trisphosphate-dependent Rac exchange factor 1 | 9.49 | 1.05E-03 |
| 8175217 | GPC4 | glypican 4 | 9.44 | 2.93E-06 |
| 8078650 | CTDSPL | CTD (carboxy-terminal domain, RNA polymerase II, polypeptide A) small phosphatase-like | 9.10 | 1.53E-07 |
| 8088491 | CADPS | Ca++-dependent secretion activator | 9.09 | 3.44E-05 |
| 7953603 | C1S | complement component 1, s subcomponent | 9.01 | 2.45E-06 |
| 8113130 | MCTP1 | multiple C2 domains, transmembrane 1 | 8.91 | 2.51E-07 |
| 7922846 | FAM129A | family with sequence similarity 129, member A | 8.72 | 1.19E-04 |
| 8006999 | CSF3 | colony stimulating factor 3 (granulocyte) | 8.52 | 2.24E-05 |
| 8090162 | ITGB5 | integrin, beta 5 | 8.46 | 1.27E-06 |
| 7904293 | PTGFRN | prostaglandin F2 receptor negative regulator | 8.39 | 2.02E-04 |
| 7906954 | PBX1 | pre-B-cell leukemia homeobox 1 | 8.22 | 4.57E-05 |
| 8031632 | ZNF542 | zinc finger protein 542 | 8.19 | 1.41E-05 |
| 8155754 | MAMDC2 | MAM domain containing 2 | 8.02 | 1.83E-04 |
| 8088415 | FAM107A | family with sequence similarity 107, member A | 7.96 | 2.18E-05 |
| 7951977 | FXYD6 | FXYD domain containing ion transport regulator 6 | 7.94 | 4.01E-04 |
| 7898057 | PDPN | podoplanin | 7.89 | 2.75E-04 |
| 8098204 | CPE | carboxypeptidase E | 7.84 | 2.25E-04 |
| 8066493 | SLPI | secretory leukocyte peptidase inhibitor | 7.78 | 9.00E-04 |
| 8023267 | MYO5B | myosin VB | 7.73 | 1.51E-05 |
| 8128123 | RRAGD | Ras-related GTP binding D | 7.48 | 2.02E-05 |
| 8054722 | IL1B | interleukin 1, beta | 7.41 | 8.92E-04 |
| 8160332 | MLLT3 | myeloid/lymphoid or mixed-lineage leukemia (trithorax homolog, Drosophila); translocated to, 3 | 7.36 | 1.53E-05 |
| 7903092 | FNBP1L | formin binding protein 1-like | 7.31 | 3.66E-04 |
| 8113666 | SEMA6A | sema domain, transmembrane domain (TM), and cytoplasmic domain, (semaphorin) 6A | 7.22 | 1.22E-05 |
| 8117054 | CAP2 | CAP, adenylate cyclase-associated protein, 2 (yeast) | 6.78 | 9.43E-08 |
| 7943998 | NNMT | nicotinamide N-methyltransferase | 6.70 | 2.02E-06 |
| 7977933 | SLC7A8 | solute carrier family 7 (amino acid transporter, L-type), member 8 | 6.62 | 5.84E-04 |
| 8130993 | FAM20C | family with sequence similarity 20, member C | 6.55 | 5.26E-04 |
| 8140358 | CCL26 | chemokine (C-C motif) ligand 26 | 6.54 | 3.39E-04 |
| 8122426 | PHACTR2 | phosphatase and actin regulator 2 | 6.46 | 8.64E-04 |
| 8174576 | AMOT | angiomotin | 6.45 | 7.47E-05 |
| 7903358 | VCAM1 | vascular cell adhesion molecule 1 | 6.43 | 5.91E-05 |
| 8051241 | ALK | anaplastic lymphoma receptor tyrosine kinase | 6.41 | 1.60E-04 |
| 8054731 | IL1F8 | interleukin 1 family, member 8 (eta) | 6.35 | 5.60E-06 |
| 8091678 | VEPH1 | ventricular zone expressed PH domain homolog 1 (zebrafish) | 6.29 | 1.15E-04 |
| 8091260 | SLC9A9 | solute carrier family 9 (sodium/hydrogen exchanger), member 9 | 6.26 | 2.37E-04 |
| 8068022 | MIR155HG | MIR155 host gene (non-protein coding) | 6.11 | 1.71E-07 |
| 8066266 | MAFB | v-maf musculoaponeurotic fibrosarcoma oncogene homolog B (avian) | 6.09 | 8.47E-05 |
| 7960874 | C3AR1 | complement component 3a receptor 1 | 6.02 | 2.61E-04 |
| 8134117 | FZD1 | frizzled homolog 1 (Drosophila) | 6.01 | 6.93E-04 |
| 8099721 | SEL1L3 | sel-1 suppressor of lin-12-like 3 (C. elegans) | 6.00 | 1.34E-04 |
| 8149448 | MSR1 | macrophage scavenger receptor 1 | 5.94 | 1.31E-03 |
| 8121319 | SOBP | sine oculis binding protein homolog (Drosophila) | 5.94 | 8.26E-05 |
| 7901720 | PRKAA2 | protein kinase, AMP-activated, alpha 2 catalytic subunit | 5.91 | 1.05E-04 |
| 8045637 | KIF5C | kinesin family member 5C | 5.88 | 7.73E-05 |
| 8095680 | IL8 | interleukin 8 | 5.88 | 6.73E-04 |
| 8166925 | MAOA | monoamine oxidase A | 5.87 | 5.61E-06 |
| 8104124 | FRG2 | FSHD region gene 2-like | 5.85 | 1.74E-06 |
| 8084880 | HES1 | hairy and enhancer of split 1, (Drosophila) | 5.79 | 1.88E-06 |
| 8154951 | GLUL | glutamate-ammonia ligase | 5.74 | 2.68E-04 |
| 8007931 | ITGB3 | integrin, beta 3 (platelet glycoprotein IIIa, antigen CD61) | 5.71 | 2.45E-05 |
| 7951703 | DRD2 | dopamine receptor D2 | 5.67 | 3.94E-04 |
| 7957386 | ACSS3 | acyl-CoA synthetase short-chain family member 3 | 5.66 | 1.56E-05 |
| 7951309 | MMP13 | matrix metallopeptidase 13 (collagenase 3) | 5.65 | 6.64E-04 |
| 8142997 | PLXNA4 | plexin A4 | 5.59 | 2.45E-04 |
| 8060963 | SNAP25 | synaptosomal-associated protein, 25kDa | 5.51 | 3.84E-05 |
| 8103745 | HAND2 | heart and neural crest derivatives expressed 2 | 5.51 | 9.49E-05 |
| 7923547 | CHI3L1 | chitinase 3-like 1 (cartilage glycoprotein-39) | 5.49 | 1.44E-04 |
| 8053551 | REEP1 | receptor accessory protein 1 | 5.41 | 8.26E-04 |
| 7965941 | GLT8D2 | glycosyltransferase 8 domain containing 2 | 5.32 | 3.72E-04 |
| 8180282 | PCDHGB4 | protocadherin gamma subfamily B, 4 | 5.25 | 7.48E-04 |
| 7933488 | C10orf72 | chromosome 10 open reading frame 72 | 5.24 | 6.26E-05 |
| 7917199 | TTLL7 | tubulin tyrosine ligase-like family, member 7 | 5.20 | 3.31E-05 |
| 8152812 | FAM84B | family with sequence similarity 84, member B | 5.18 | 5.50E-05 |
| 7961891 | BHLHE41 | basic helix-loop-helix family, member e41 | 5.16 | 6.97E-04 |
| 7937251 | FRG2 | FSHD region gene 2-like family | 5.16 | 8.96E-06 |
| 7918157 | VAV3 | vav 3 guanine nucleotide exchange factor | 5.04 | 8.13E-06 |
| 7960744 | C1R | complement component 1, r subcomponent | 5.04 | 1.03E-04 |
| 8050427 | FAM49A | family with sequence similarity 49, member A | 4.96 | 8.24E-05 |
| 8149825 | STC1 | stanniocalcin 1 | 4.95 | 1.55E-05 |
| 8146533 | FAM110B | family with sequence similarity 110, member B | 4.95 | 3.45E-04 |
| 7952205 | MCAM | melanoma cell adhesion molecule | 4.92 | 3.83E-04 |
| 8056860 | WIPF1 | WAS/WASL interacting protein family, member 1 | 4.91 | 5.58E-07 |
| 8033257 | C3 | complement component 3 | 4.91 | 7.06E-05 |
| 7972055 | KCTD12 | potassium channel tetramerisation domain containing 12 | 4.83 | 4.09E-05 |
| 8139207 | INHBA | inhibin, beta A | 4.82 | 1.13E-05 |
| 7913655 | ID3 | inhibitor of DNA binding 3, dominant negative helix-loop-helix protein | 4.77 | 3.41E-05 |
| 8068671 | BACE2 | beta-site APP-cleaving enzyme 2 | 4.77 | 5.37E-04 |
| 7944722 | UBASH3B | ubiquitin associated and SH3 domain containing B | 4.72 | 5.36E-04 |
| 8133860 | GNAI1 | guanine nucleotide binding protein (G protein), alpha inhibiting activity polypeptide 1 | 4.68 | 6.49E-04 |
| 8113234 | PCSK1 | proprotein convertase subtilisin/kexin type 1 | 4.66 | 5.77E-04 |
| 7921882 | OLFML2B | olfactomedin-like 2B | 4.63 | 3.21E-04 |
| 7924987 | AGT | angiotensinogen (serpin peptidase inhibitor, clade A, member 8) | 4.60 | 1.05E-04 |
| 7989365 | RORA | RAR-related orphan receptor A | 4.57 | 3.22E-04 |
| 8140478 | PION | pigeon homolog (Drosophila) | 4.56 | 6.80E-08 |
| 7974366 | PTGER2 | prostaglandin E receptor 2 (subtype EP2), 53kDa | 4.51 | 4.69E-06 |
| 7944751 | C11orf63 | chromosome 11 open reading frame 63 | 4.49 | 1.03E-04 |
| 8152703 | FBXO32 | F-box protein 32 | 4.45 | 3.84E-04 |
| 7914580 | FNDC5 | fibronectin type III domain containing 5 | 4.43 | 1.33E-04 |
| 8108744 | PCDHB14 | protocadherin beta 14 | 4.41 | 1.12E-04 |
| 7925062 | SIPA1L2 | signal-induced proliferation-associated 1 like 2 | 4.39 | 2.41E-04 |
| 8108733 | PCDHB12 | protocadherin beta 12 | 4.39 | 1.66E-04 |
| 8157524 | TLR4 | toll-like receptor 4 | 4.33 | 7.29E-04 |
| 8057578 | CALCRL | calcitonin receptor-like | 4.31 | 2.73E-05 |
| 8114287 | SPOCK1 | sparc/osteonectin, cwcv and kazal-like domains proteoglycan (testican) 1 | 4.31 | 6.04E-04 |
| 8023828 | NETO1 | neuropilin (NRP) and tolloid (TLL)-like 1 | 4.25 | 6.99E-05 |
| 8086600 | CCR1 | chemokine (C-C motif) receptor 1 | 4.23 | 4.93E-05 |
| 8099633 | PPARGC1A | peroxisome proliferator-activated receptor gamma, coactivator 1 alpha | 4.20 | 7.62E-08 |
| 8033767 | ZNF560 | zinc finger protein 560 | 4.17 | 5.21E-05 |
| 8163202 | SVEP1 | sushi, von Willebrand factor type A, EGF and pentraxin domain containing 1 | 4.15 | 4.07E-05 |
| 8074606 | USP18/41 | ubiquitin specific peptidase 18/41 | 4.13 | 1.10E-04 |
| 8172425 | SLC38A5 | solute carrier family 38, member 5 | 4.13 | 2.22E-04 |
| 8023121 | ST8SIA5 | ST8 alpha-N-acetyl-neuraminide alpha-2,8-sialyltransferase 5 | 4.13 | 1.66E-05 |
| 7908459 | CFH | complement factor H | 4.12 | 3.50E-05 |
| 7958913 | OAS2 | 2'-5'-oligoadenylate synthetase 2, 69/71kDa | 4.09 | 1.41E-03 |
| 7945663 | LOC402778 | CD225 family protein FLJ76511 | 4.08 | 1.68E-04 |
| 8043995 | IL1R1 | interleukin 1 receptor, type I | 4.07 | 3.06E-04 |
| 7939559 | TSPAN18 | tetraspanin 18 | 4.00 | 1.66E-05 |
| 7904158 | OLFML3 | olfactomedin-like 3 | 3.99 | 6.93E-04 |
| 8115490 | ADAM19 | ADAM metallopeptidase domain 19 | 3.98 | 2.79E-04 |
| 7922689 | GLUL | glutamate-ammonia ligase | 3.96 | 1.42E-04 |
| 8044605 | LOC654433 | hypothetical LOC654433 | 3.95 | 2.39E-04 |
| 7970441 | GJB2 | gap junction protein, beta 2, 26kDa | 3.93 | 3.54E-05 |
| 8045889 | TANC1 | tetratricopeptide repeat, ankyrin repeat and coiled-coil containing 1 | 3.92 | 1.70E-05 |
| 8081219 | ST3GAL6 | ST3 beta-galactoside alpha-2,3-sialyltransferase 6 | 3.91 | 4.88E-04 |
| 8088458 | FHIT | fragile histidine triad gene | 3.89 | 5.92E-04 |
| 8111569 | RANBP3L | RAN binding protein 3-like | 3.85 | 1.30E-03 |
| 8100870 | ADAMTS3 | ADAM metallopeptidase with thrombospondin type 1 motif, 3 | 3.84 | 1.65E-05 |
| 8020973 | FHOD3 | formin homology 2 domain containing 3 | 3.84 | 4.85E-05 |
| 8147756 | BAALC | brain and acute leukemia, cytoplasmic | 3.82 | 2.12E-05 |
| 8071155 | USP18 | ubiquitin specific peptidase 18 | 3.82 | 5.83E-04 |
| 7902810 | LMO4 | LIM domain only 4 | 3.81 | 2.72E-04 |
| 8039593 | ZNF667 | zinc finger protein 667 | 3.79 | 5.95E-04 |
| 8086607 | LTF | lactotransferrin | 3.76 | 1.99E-05 |
| 7984704 | NEO1 | neogenin 1 | 3.75 | 1.24E-03 |
| 8088919 | ROBO1 | roundabout, axon guidance receptor, homolog 1 (Drosophila) | 3.75 | 6.56E-05 |
| 8094751 | CHRNA9 | cholinergic receptor, nicotinic, alpha 9 | 3.73 | 6.33E-05 |
| 8002882 | CHST6 | carbohydrate (N-acetylglucosamine 6-O) sulfotransferase 6 | 3.72 | 7.65E-04 |
| 8083616 | MLF1 | myeloid leukemia factor 1 | 3.72 | 9.17E-04 |
| 8056323 | FIGN | fidgetin | 3.70 | 1.07E-05 |
| 7923534 | MYBPH | myosin binding protein H | 3.66 | 2.20E-05 |
| 7946446 | NRIP3 | nuclear receptor interacting protein 3 | 3.64 | 1.08E-03 |
| 7918657 | PTPN22 | protein tyrosine phosphatase, non-receptor type 22 (lymphoid) | 3.64 | 5.91E-06 |
| 7909789 | TGFB2 | transforming growth factor, beta 2 | 3.64 | 4.68E-05 |
| 7980908 | FBLN5 | fibulin 5 | 3.64 | 8.91E-04 |
| 8149324 | FAM167A | family with sequence similarity 167, member A | 3.63 | 3.48E-04 |
| 8108737 | PCDHB13 | protocadherin beta 13 | 3.61 | 9.93E-05 |
| 8099967 | RBM47 | RNA binding motif protein 47 | 3.61 | 1.23E-03 |
| 7948588 | SYT7 | synaptotagmin VII | 3.61 | 2.06E-04 |
| 8086517 | CDCP1 | CUB domain containing protein 1 | 3.59 | 3.53E-04 |
| 8094778 | UCHL1 | ubiquitin carboxyl-terminal esterase L1 (ubiquitin thiolesterase) | 3.58 | 1.91E-04 |
| 7965410 | DCN | decorin | 3.58 | 4.12E-04 |
| 8108716 | PCDHB16 | protocadherin beta 16 | 3.57 | 1.80E-04 |
| 8041644 | PLEKHH2 | pleckstrin homology domain containing, family H (with MyTH4 domain) member 2 | 3.54 | 8.35E-05 |
| 8083599 | C3orf55 | chromosome 3 open reading frame 55 | 3.54 | 2.68E-05 |
| 7953284 | NTF3 | neurotrophin 3 | 3.54 | 9.49E-04 |
| 8067270 | APCDD1L | adenomatosis polyposis coli down-regulated 1-like | 3.53 | 4.82E-06 |
| 8114797 | SPRY4 | sprouty homolog 4 (Drosophila) | 3.51 | 2.94E-04 |
| 8063382 | SNAI1 | snail homolog 1 (Drosophila) | 3.50 | 1.38E-07 |
| 8039484 | IL11 | interleukin 11 | 3.50 | 4.54E-04 |
| 8068583 | KCNJ15 | potassium inwardly-rectifying channel, subfamily J, member 15 | 3.50 | 3.30E-05 |
| 8023220 | SMAD7 | SMAD family member 7 | 3.49 | 2.38E-04 |
| 7950701 | ODZ4 | odz, odd Oz/ten-m homolog 4 (Drosophila) | 3.49 | 6.18E-04 |
| 8084206 | B3GNT5 | UDP-GlcNAc:betaGal beta-1,3-N-acetylglucosaminyltransferase 5 | 3.49 | 9.18E-04 |
| 8108706 | PCDHB17 | protocadherin beta 17 pseudogene | 3.48 | 5.40E-04 |
| 8096733 | SGMS2 | sphingomyelin synthase 2 | 3.45 | 3.26E-04 |
| 8154692 | TEK | TEK tyrosine kinase, endothelial | 3.45 | 1.17E-04 |
| 8096511 | BMPR1B | bone morphogenetic protein receptor, type IB | 3.43 | 1.59E-04 |
| 8120279 | LRRC1 | leucine rich repeat containing 1 | 3.43 | 6.08E-04 |
| 8129888 | NHSL1 | NHS-like 1 | 3.41 | 1.41E-04 |
| 8069269 | COL6A1 | collagen, type VI, alpha 1 | 3.41 | 4.89E-04 |
| 7917779 | GCLM | glutamate-cysteine ligase, modifier subunit | 3.41 | 3.12E-07 |
| 8041206 | LBH | limb bud and heart development homolog (mouse) | 3.40 | 1.54E-04 |
| 7976560 | BDKRB2 | bradykinin receptor B2 | 3.40 | 4.75E-04 |
| 7928147 | ADAMTS14 | ADAM metallopeptidase with thrombospondin type 1 motif, 14 | 3.38 | 3.55E-05 |
| 8156770 | GALNT12 | UDP-N-acetyl-alpha-D-galactosamine:polypeptide N-acetylgalactosaminyltransferase 12 (GalNAc-T12) | 3.34 | 6.08E-04 |
| 8108713 | PCDHB8 | protocadherin beta 8 | 3.33 | 3.48E-04 |
| 8027002 | GDF15 | growth differentiation factor 15 | 3.32 | 9.89E-04 |
| 8131600 | TSPAN13 | tetraspanin 13 | 3.32 | 4.97E-04 |
| 8099685 | LGI2 | leucine-rich repeat LGI family, member 2 | 3.32 | 1.23E-03 |
| 8108724 | PCDHB10 | protocadherin beta 10 | 3.31 | 5.55E-04 |
| 8160452 | CDKN2B | cyclin-dependent kinase inhibitor 2B (p15, inhibits CDK4) | 3.31 | 1.06E-03 |
| 8006433 | CCL2 | chemokine (C-C motif) ligand 2 | 3.31 | 4.09E-04 |
| 8068238 | IFNAR2 | interferon (alpha, beta and omega) receptor 2 | 3.30 | 7.31E-04 |
| 8171034 | SPRY3 | sprouty homolog 3 (Drosophila) | 3.29 | 6.95E-04 |
| 8176955 | SPRY3 | sprouty homolog 3 (Drosophila) | 3.29 | 6.95E-04 |
| 8070182 | RCAN1 | regulator of calcineurin 1 | 3.28 | 1.19E-03 |
| 8121850 | HEY2 | hairy/enhancer-of-split related with YRPW motif 2 | 3.28 | 2.51E-04 |
| 7968577 | NBEA | neurobeachin | 3.28 | 3.05E-04 |
| 8108720 | PCDHB9 | protocadherin beta 9 | 3.27 | 2.35E-04 |
| 8122396 | AIG1 | androgen-induced 1 | 3.26 | 1.21E-03 |
| 7923233 | KIF21B | kinesin family member 21B | 3.26 | 1.01E-04 |
| 8111387 | ADAMTS12 | ADAM metallopeptidase with thrombospondin type 1 motif, 12 | 3.25 | 3.84E-04 |
| 7974902 | RHOJ | ras homolog gene family, member J | 3.25 | 5.82E-04 |
| 7975459 | SIPA1L1 | signal-induced proliferation-associated 1 like 1 | 3.24 | 1.09E-03 |
| 8022559 | ANKRD29 | ankyrin repeat domain 29 | 3.21 | 1.95E-04 |
| 7899562 | PTPRU | protein tyrosine phosphatase, receptor type, U | 3.21 | 3.69E-04 |
| 7957072 | RAB3IP | RAB3A interacting protein (rabin3) | 3.21 | 6.46E-05 |
| 8108217 | TGFBI | transforming growth factor, beta-induced, 68kDa | 3.20 | 9.20E-05 |
| 8056837 | GPR155 | G protein-coupled receptor 155 | 3.20 | 9.13E-04 |
| 8140971 | SAMD9L | sterile alpha motif domain containing 9-like | 3.19 | 7.24E-04 |
| 8072314 | MGC20647 | hypothetical protein MGC20647 | 3.19 | 4.60E-04 |
| 8077786 | IRAK2 | interleukin-1 receptor-associated kinase 2 | 3.18 | 5.68E-04 |
| 8050007 | PXDN | peroxidasin homolog (Drosophila) | 3.17 | 8.13E-07 |
| 8043945 | MAP4K4 | mitogen-activated protein kinase kinase kinase kinase 4 | 3.17 | 7.79E-04 |
| 8101992 | SLC39A8 | solute carrier family 39 (zinc transporter), member 8 | 3.16 | 1.86E-04 |
| 8130867 | THBS2 | thrombospondin 2 | 3.15 | 6.42E-05 |
| 8083214 | CHST2 | carbohydrate (N-acetylglucosamine-6-O) sulfotransferase 2 | 3.12 | 2.70E-04 |
| 7917516 | GBP1P1 | guanylate binding protein 1, interferon-inducible pseudogene 1 | 3.12 | 3.52E-06 |
| 7917516 | GBP1 | guanylate binding protein 1, interferon-inducible | 3.12 | 3.52E-06 |
| 8134051 | C7orf63 | chromosome 7 open reading frame 63 | 3.12 | 1.23E-03 |
| 8088485 | FEZF2 | FEZ family zinc finger 2 | 3.11 | 8.00E-04 |
| 8057990 | ANKRD44 | ankyrin repeat domain 44 | 3.11 | 2.56E-04 |
| 8060418 | SIRPA | signal-regulatory protein alpha | 3.11 | 1.31E-03 |
| 8080964 | GXYLT2 | glucoside xylosyltransferase 2 | 3.10 | 3.50E-05 |
| 7971461 | LCP1 | lymphocyte cytosolic protein 1 (L-plastin) | 3.09 | 2.43E-05 |
| 8069301 | COL6A2 | collagen, type VI, alpha 2 | 3.09 | 1.06E-03 |
| 7985248 | KIAA1024 | KIAA1024 | 3.08 | 9.55E-04 |
| 7922807 | GLT25D2 | glycosyltransferase 25 domain containing 2 | 3.07 | 2.03E-05 |
| 8112940 | SSBP2 | single-stranded DNA binding protein 2 | 3.06 | 4.90E-07 |
| 7965335 | DUSP6 | dual specificity phosphatase 6 | 3.06 | 6.31E-04 |
| 8153002 | NDRG1 | N-myc downstream regulated 1 | 3.04 | 3.48E-04 |
| 7903786 | CSF1 | colony stimulating factor 1 (macrophage) | 3.03 | 3.16E-06 |
| 8029489 | BCAM | basal cell adhesion molecule (Lutheran blood group) | 3.03 | 1.32E-03 |
| 8138728 | HOXA4 | homeobox A4 | 3.02 | 3.07E-04 |
| 7900146 | ZC3H12A | zinc finger CCCH-type containing 12A | 3.01 | 1.07E-04 |
| 8124848 | IER3 | immediate early response 3 | 3.00 | 1.31E-05 |
| 8179704 | IER3 | immediate early response 3 | 3.00 | 1.31E-05 |
| 8096959 | ANK2 | ankyrin 2, neuronal | 3.00 | 1.78E-04 |
| 8147145 | ATP6V0D2 | ATPase, H+ transporting, lysosomal 38kDa, V0 subunit d2 | 2.99 | 1.87E-05 |
| 8084951 | LRRC33 | leucine rich repeat containing 33 | 2.99 | 3.45E-05 |
| 8085984 | OSBPL10 | oxysterol binding protein-like 10 | 2.99 | 5.35E-04 |
| 8173059 | WNK3 | WNK lysine deficient protein kinase 3 | 2.98 | 1.56E-05 |
| 8091327 | PLSCR1 | phospholipid scramblase 1 | 2.97 | 6.61E-05 |
| 8078227 | KAT2B | K(lysine) acetyltransferase 2B | 2.97 | 6.05E-04 |
| 7898448 | PADI4 | peptidyl arginine deiminase, type IV | 2.96 | 1.05E-04 |
| 8136341 | BPGM | 2,3-bisphosphoglycerate mutase | 2.95 | 2.24E-04 |
| 7997336 | VAT1L | vesicle amine transport protein 1 homolog (T. californica)-like | 2.95 | 2.77E-04 |
| 8096335 | HERC6 | hect domain and RLD 6 | 2.95 | 1.27E-03 |
| 8081386 | NFKBIZ | nuclear factor of kappa light polypeptide gene enhancer in B-cells inhibitor, zeta | 2.93 | 4.12E-05 |
| 7926545 | PLXDC2 | plexin domain containing 2 | 2.93 | 6.85E-05 |
| 8123658 | SLC22A23 | solute carrier family 22, member 23 | 2.93 | 8.18E-04 |
| 8140504 | MAGI2 | membrane associated guanylate kinase, WW and PDZ domain containing 2 | 2.92 | 4.97E-04 |
| 7909027 | NFASC | neurofascin | 2.91 | 1.40E-03 |
| 8040090 | RNF144A | ring finger protein 144A | 2.91 | 1.12E-03 |
| 8139488 | IGFBP3 | insulin-like growth factor binding protein 3 | 2.91 | 5.89E-04 |
| 7914127 | IFI6 | interferon, alpha-inducible protein 6 | 2.91 | 1.24E-03 |
| 8178435 | IER3 | immediate early response 3 | 2.89 | 1.03E-05 |
| 8100977 | CXCL5 | chemokine (C-X-C motif) ligand 5 | 2.89 | 9.42E-05 |
| 8165974 | CLCN4 | chloride channel 4 | 2.89 | 8.55E-05 |
| 7902702 | CLCA2 | chloride channel accessory 2 | 2.89 | 3.98E-04 |
| 8085665 | RFTN1 | raftlin, lipid raft linker 1 | 2.87 | 5.42E-04 |
| 8029465 | BCL3 | B-cell CLL/lymphoma 3 | 2.85 | 8.68E-04 |
| 7931353 | PTPRE | protein tyrosine phosphatase, receptor type, E | 2.83 | 1.38E-03 |
| 8114572 | HBEGF | heparin-binding EGF-like growth factor | 2.83 | 4.15E-07 |
| 8113761 | ZNF608 | zinc finger protein 608 | 2.83 | 1.09E-03 |
| 8010967 | NXN | nucleoredoxin | 2.83 | 4.85E-04 |
| 8149865 | EBF2 | early B-cell factor 2 | 2.82 | 2.19E-04 |
| 8083075 | ACPL2 | acid phosphatase-like 2 | 2.82 | 2.96E-04 |
| 7903959 | C1orf88 | chromosome 1 open reading frame 88 | 2.81 | 1.21E-04 |
| 8039054 | ZNF347 | zinc finger protein 347 | 2.81 | 6.91E-05 |
| 8143961 | PRKAG2 | protein kinase, AMP-activated, gamma 2 non-catalytic subunit | 2.81 | 4.77E-04 |
| 7928189 | UNC5B | unc-5 homolog B (C. elegans) | 2.80 | 3.06E-04 |
| 7936968 | ADAM12 | ADAM metallopeptidase domain 12 | 2.80 | 4.43E-04 |
| 7919800 | CTSS | cathepsin S | 2.79 | 8.16E-04 |
| 8005661 | SPECC1 | sperm antigen with calponin homology and coiled-coil domains 1 | 2.77 | 4.17E-04 |
| 8130556 | SOD2 | superoxide dismutase 2, mitochondrial | 2.77 | 1.14E-04 |
| 7922717 | RGS16 | regulator of G-protein signaling 16 | 2.76 | 2.45E-05 |
| 8056257 | FAP | fibroblast activation protein, alpha | 2.76 | 1.19E-03 |
| 7988763 | TNFAIP8L3 | tumor necrosis factor, alpha-induced protein 8-like 3 | 2.75 | 5.11E-04 |
| 8153021 | ST3GAL1 | ST3 beta-galactoside alpha-2,3-sialyltransferase 1 | 2.75 | 8.39E-04 |
| 8083749 | PPM1L | protein phosphatase, Mg2+/Mn2+ dependent, 1L | 2.75 | 1.24E-03 |
| 8104930 | SLC1A3 | solute carrier family 1 (glial high affinity glutamate transporter), member 3 | 2.74 | 5.24E-05 |
| 8044391 | MERTK | c-mer proto-oncogene tyrosine kinase | 2.74 | 9.82E-04 |
| 8016646 | COL1A1 | collagen, type I, alpha 1 | 2.72 | 2.62E-04 |
| 7933733 | FAM13C | family with sequence similarity 13, member C | 2.70 | 5.34E-04 |
| 8066925 | PTGIS | prostaglandin I2 (prostacyclin) synthase | 2.69 | 4.30E-04 |
| 8069565 | BTG3 | BTG family, member 3 | 2.69 | 5.65E-06 |
| 8061406 | TMEM90B | transmembrane protein 90B | 2.68 | 2.92E-04 |
| 7954631 | FAR2 | fatty acyl CoA reductase 2 | 2.68 | 7.53E-04 |
| 7903920 | CHI3L2 | chitinase 3-like 2 | 2.67 | 1.34E-06 |
| 8105411 | IL31RA | interleukin 31 receptor A | 2.67 | 7.86E-04 |
| 7977046 | TNFAIP2 | tumor necrosis factor, alpha-induced protein 2 | 2.67 | 6.89E-04 |
| 8166714 | LANCL3 | LanC lantibiotic synthetase component C-like 3 (bacterial) | 2.65 | 6.87E-04 |
| 8105077 | CARD6 | caspase recruitment domain family, member 6 | 2.65 | 7.02E-04 |
| 7975390 | SMOC1 | SPARC related modular calcium binding 1 | 2.65 | 6.65E-04 |
| 7965964 | SLC41A2 | solute carrier family 41, member 2 | 2.65 | 2.06E-05 |
| 7926223 | CAMK1D | calcium/calmodulin-dependent protein kinase ID | 2.64 | 2.43E-04 |
| 8066985 | FAM65C | family with sequence similarity 65, member C | 2.64 | 2.67E-04 |
| 7953040 | CACNA1C | calcium channel, voltage-dependent, L type, alpha 1C subunit | 2.63 | 8.13E-06 |
| 7915392 | HIVEP3 | human immunodeficiency virus type I enhancer binding protein 3 | 2.62 | 2.41E-04 |
| 8135378 | PRKAR2B | protein kinase, cAMP-dependent, regulatory, type II, beta | 2.62 | 7.10E-04 |
| 8096361 | HERC5 | hect domain and RLD 5 | 2.62 | 3.99E-04 |
| 8175016 | APLN | apelin | 2.61 | 7.96E-04 |
| 8090988 | CEP70 | centrosomal protein 70kDa | 2.60 | 1.14E-03 |
| 8070538 | C2CD2 | C2 calcium-dependent domain containing 2 | 2.60 | 9.75E-05 |
| 8159900 | GLIS3 | GLIS family zinc finger 3 | 2.60 | 2.42E-07 |
| 8089701 | ZBTB20 | zinc finger and BTB domain containing 20 | 2.60 | 1.37E-03 |
| 7976567 | BDKRB1 | bradykinin receptor B1 | 2.60 | 1.59E-05 |
| 8166447 | PTCHD1 | patched domain containing 1 | 2.59 | 2.96E-05 |
| 7940022 | RTN4RL2 | reticulon 4 receptor-like 2 | 2.59 | 9.73E-07 |
| 8082314 | PLXNA1 | plexin A1 | 2.58 | 9.30E-04 |
| 8090018 | PARP9 | poly (ADP-ribose) polymerase family, member 9 | 2.58 | 2.67E-04 |
| 7974920 | SYNE2 | spectrin repeat containing, nuclear envelope 2 | 2.56 | 8.19E-04 |
| 8023696 | SERPINB3 | serpin peptidase inhibitor, clade B (ovalbumin), member 3 | 2.56 | 1.16E-04 |
| 8061564 | ID1 | inhibitor of DNA binding 1, dominant negative helix-loop-helix protein | 2.54 | 3.44E-04 |
| 7901054 | PLK3 | polo-like kinase 3 | 2.54 | 4.63E-04 |
| 8140113 | STX1A | syntaxin 1A (brain) | 2.53 | 5.70E-04 |
| 8067530 | C20orf200 | chromosome 20 open reading frame 200 | 2.53 | 9.41E-05 |
| 8031650 | ZNF471 | zinc finger protein 471 | 2.52 | 1.13E-03 |
| 7898809 | EPHB2 | EPH receptor B2 | 2.52 | 6.04E-04 |
| 8019988 | PTPRM | protein tyrosine phosphatase, receptor type, M | 2.52 | 1.41E-03 |
| 8110055 | CPEB4 | cytoplasmic polyadenylation element binding protein 4 | 2.52 | 5.82E-04 |
| 8150002 | ZNF395 | zinc finger protein 395 | 2.51 | 6.72E-04 |
| 8150002 | FBXO16 | F-box protein 16 | 2.51 | 6.72E-04 |
| 7964834 | CPM | carboxypeptidase M | 2.50 | 1.32E-03 |
| 8108370 | EGR1 | early growth response 1 | 2.50 | 6.94E-04 |
| 8070876 | POFUT2 | protein O-fucosyltransferase 2 | 2.49 | 3.03E-04 |
| 8109305 | SYNPO | synaptopodin | 2.48 | 7.43E-04 |
| 7916616 | CYP2J2 | cytochrome P450, family 2, subfamily J, polypeptide 2 | 2.48 | 4.40E-04 |
| 8025402 | ANGPTL4 | angiopoietin-like 4 | 2.48 | 1.48E-04 |
| 7954293 | PDE3A | phosphodiesterase 3A, cGMP-inhibited | 2.47 | 2.50E-04 |
| 7902687 | CYR61 | cysteine-rich, angiogenic inducer, 61 | 2.47 | 8.08E-04 |
| 8136336 | AKR1B10 | aldo-keto reductase family 1, member B10 (aldose reductase) | 2.47 | 8.51E-04 |
| 8063437 | TSHZ2 | teashirt zinc finger homeobox 2 | 2.47 | 2.91E-04 |
| 7918323 | SORT1 | sortilin 1 | 2.46 | 1.66E-04 |
| 8065280 | RALGAPA2 | Ral GTPase activating protein, alpha subunit 2 (catalytic) | 2.46 | 8.29E-04 |
| 7948332 | LPXN | leupaxin | 2.46 | 4.97E-05 |
| 8067040 | NFATC2 | nuclear factor of activated T-cells, cytoplasmic, calcineurin-dependent 2 | 2.46 | 2.17E-04 |
| 8129861 | IFNGR1 | interferon gamma receptor 1 | 2.45 | 9.91E-04 |
| 7974372 | GPR137C | G protein-coupled receptor 137C | 2.45 | 2.71E-04 |
| 8157487 | PAPPA | pregnancy-associated plasma protein A, pappalysin 1 | 2.44 | 1.08E-03 |
| 8089015 | PROS1 | protein S (alpha) | 2.44 | 1.49E-07 |
| 8060805 | CHGB | chromogranin B (secretogranin 1) | 2.44 | 7.45E-05 |
| 8088550 | PRICKLE2 | prickle homolog 2 (Drosophila) | 2.43 | 6.99E-06 |
| 7946292 | CYB5R2 | cytochrome b5 reductase 2 | 2.43 | 3.32E-04 |
| 8082165 | KALRN | kalirin, RhoGEF kinase | 2.43 | 4.37E-05 |
| 8117045 | RBM24 | RNA binding motif protein 24 | 2.42 | 3.27E-04 |
| 8177628 | CCDC125 | coiled-coil domain containing 125 | 2.41 | 1.23E-03 |
| 8005097 | HS3ST3B1 | heparan sulfate (glucosamine) 3-O-sulfotransferase 3B1 | 2.41 | 1.25E-03 |
| 8113796 | FLJ44606 | glutaredoxin-like protein YDR286C homolog | 2.41 | 1.03E-03 |
| 8083318 | LOC646903 | hypothetical LOC646903 | 2.41 | 1.09E-04 |
| 8091283 | PLOD2 | procollagen-lysine, 2-oxoglutarate 5-dioxygenase 2 | 2.40 | 8.46E-05 |
| 7956856 | MSRB3 | methionine sulfoxide reductase B3 | 2.40 | 3.94E-05 |
| 7908125 | RGL1 | ral guanine nucleotide dissociation stimulator-like 1 | 2.38 | 5.37E-04 |
| 8024111 | CNN2 | calponin 2 | 2.37 | 9.11E-04 |
| 8161701 | TMEM2 | transmembrane protein 2 | 2.36 | 1.19E-03 |
| 8041383 | LTBP1 | latent transforming growth factor beta binding protein 1 | 2.36 | 3.29E-04 |
| 8139500 | TNS3 | tensin 3 | 2.36 | 5.81E-05 |
| 8082100 | PARP14 | poly (ADP-ribose) polymerase family, member 14 | 2.34 | 8.13E-04 |
| 8163109 | C9orf4 | chromosome 9 open reading frame 4 | 2.34 | 9.65E-04 |
| 8123562 | GMDS | GDP-mannose 4,6-dehydratase | 2.34 | 4.99E-04 |
| 8128247 | BACH2 | BTB and CNC homology 1, basic leucine zipper transcription factor 2 | 2.34 | 1.04E-03 |
| 8098263 | PALLD | palladin, cytoskeletal associated protein | 2.34 | 1.89E-04 |
| 8116418 | GFPT2 | glutamine-fructose-6-phosphate transaminase 2 | 2.32 | 3.05E-05 |
| 8155200 | CCIN | calicin | 2.32 | 6.05E-04 |
| 8069689 | ADAMTS5 | ADAM metallopeptidase with thrombospondin type 1 motif, 5 | 2.32 | 4.52E-05 |
| 8069178 | ADARB1 | adenosine deaminase, RNA-specific, B1 | 2.31 | 7.66E-04 |
| 8138689 | SKAP2 | src kinase associated phosphoprotein 2 | 2.31 | 1.04E-03 |
| 8029331 | ZNF221 | zinc finger protein 221 | 2.31 | 1.15E-03 |
| 8021365 | LOC100505549 | hypothetical LOC100505549 | 2.31 | 7.56E-04 |
| 8174313 | MORC4 | MORC family CW-type zinc finger 4 | 2.31 | 1.69E-06 |
| 8098611 | TLR3 | toll-like receptor 3 | 2.30 | 1.38E-03 |
| 8077490 | LMCD1 | LIM and cysteine-rich domains 1 | 2.30 | 3.96E-04 |
| 8143054 | AKR1B1 | aldo-keto reductase family 1, member B1 (aldose reductase) | 2.30 | 2.29E-04 |
| 7924499 | TLR5 | toll-like receptor 5 | 2.29 | 4.42E-04 |
| 7965166 | PPFIA2 | protein tyrosine phosphatase, receptor type, f polypeptide (PTPRF), interacting protein (liprin), alpha 2 | 2.28 | 5.45E-04 |
| 7974870 | SNAPC1 | small nuclear RNA activating complex, polypeptide 1, 43kDa | 2.27 | 5.13E-04 |
| 8116534 | TRIM52 | tripartite motif containing 52 | 2.26 | 4.44E-05 |
| 8098041 | TMEM144 | transmembrane protein 144 | 2.26 | 3.26E-04 |
| 8019964 | ARHGAP28 | Rho GTPase activating protein 28 | 2.25 | 8.12E-04 |
| 7979158 | TXNDC16 | thioredoxin domain containing 16 | 2.25 | 8.81E-05 |
| 7908003 | NPL | N-acetylneuraminate pyruvate lyase (dihydrodipicolinate synthase) | 2.25 | 1.34E-03 |
| 8080781 | PXK | PX domain containing serine/threonine kinase | 2.24 | 1.03E-04 |
| 8172225 | EFHC2 | EF-hand domain (C-terminal) containing 2 | 2.24 | 1.18E-04 |
| 8072108 | ASPHD2 | aspartate beta-hydroxylase domain containing 2 | 2.24 | 1.14E-04 |
| 7979108 | TRIM9 | tripartite motif containing 9 | 2.23 | 3.99E-04 |
| 7978644 | NFKBIA | nuclear factor of kappa light polypeptide gene enhancer in B-cells inhibitor, alpha | 2.23 | 3.81E-04 |
| 7918379 | GSTM3 | glutathione S-transferase mu 3 (brain) | 2.22 | 6.78E-04 |
| 8174322 | MORC4 | MORC family CW-type zinc finger 4 | 2.22 | 1.43E-06 |
| 8136347 | CALD1 | caldesmon 1 | 2.22 | 3.32E-04 |
| 8078405 | CMTM7 | CKLF-like MARVEL transmembrane domain containing 7 | 2.22 | 9.38E-04 |
| 8089329 | MYH15 | myosin, heavy chain 15 | 2.22 | 9.40E-04 |
| 8083876 | SKIL | SKI-like oncogene | 2.22 | 2.24E-05 |
| 8068280 | IFNGR2 | interferon gamma receptor 2 (interferon gamma transducer 1) | 2.22 | 4.64E-04 |
| 7984364 | SMAD3 | SMAD family member 3 | 2.22 | 4.38E-04 |
| 7938396 | AMPD3 | adenosine monophosphate deaminase 3 | 2.21 | 7.98E-04 |
| 8070239 | HLCS | holocarboxylase synthetase (biotin-(proprionyl-CoA-carboxylase (ATP-hydrolysing)) ligase) | 2.21 | 9.44E-04 |
| 7985192 | AGPHD1 | aminoglycoside phosphotransferase domain containing 1 | 2.21 | 5.40E-04 |
| 8091422 | WWTR1 | WW domain containing transcription regulator 1 | 2.21 | 9.08E-04 |
| 7939383 | PRR5L | proline rich 5 like | 2.20 | 5.36E-04 |
| 8180405 | ARL4A | ADP-ribosylation factor-like 4A | 2.20 | 4.76E-05 |
| 8077728 | LOC442075 | hypothetical LOC442075 | 2.20 | 2.16E-04 |
| 8089759 | TMEM39A | transmembrane protein 39A | 2.20 | 1.42E-04 |
| 8143441 | KIAA1147 | KIAA1147 | 2.20 | 1.93E-04 |
| 8169186 | TBC1D8B | TBC1 domain family, member 8B (with GRAM domain) | 2.20 | 2.95E-04 |
| 8152215 | KLF10 | Kruppel-like factor 10 | 2.19 | 1.20E-04 |
| 8091446 | PFN2 | profilin 2 | 2.19 | 1.19E-03 |
| 8109407 | GALNT10 | UDP-N-acetyl-alpha-D-galactosamine:polypeptide N-acetylgalactosaminyltransferase 10 (GalNAc-T10) | 2.19 | 9.87E-04 |
| 8088264 | IL17RD | interleukin 17 receptor D | 2.19 | 5.02E-04 |
| 8068361 | SLC5A3 | solute carrier family 5 (sodium/myo-inositol cotransporter), member 3 | 2.19 | 1.78E-04 |
| 8135990 | FLNC | filamin C, gamma | 2.18 | 1.41E-03 |
| 8148070 | COL14A1 | collagen, type XIV, alpha 1 | 2.17 | 5.98E-04 |
| 8105040 | OSMR | oncostatin M receptor | 2.17 | 3.10E-04 |
| 8094911 | ATP10D | ATPase, class V, type 10D | 2.17 | 5.23E-04 |
| 8073088 | APOBEC3G | apolipoprotein B mRNA editing enzyme, catalytic polypeptide-like 3G | 2.17 | 5.17E-04 |
| 8069532 | HSPA13 | heat shock protein 70kDa family, member 13 | 2.16 | 6.92E-06 |
| 8030931 | ZNF528 | zinc finger protein 528 | 2.16 | 1.31E-03 |
| 7914667 | CSMD2 | CUB and Sushi multiple domains 2 | 2.16 | 3.72E-04 |
| 8031659 | ZFP28 | zinc finger protein 28 homolog (mouse) | 2.16 | 5.40E-04 |
| 8068478 | CHAF1B | chromatin assembly factor 1, subunit B (p60) | 2.16 | 7.59E-04 |
| 8089627 | SPICE1 | spindle and centriole associated protein 1 | 2.15 | 4.38E-04 |
| 8122099 | ENPP1 | ectonucleotide pyrophosphatase/phosphodiesterase 1 | 2.15 | 5.62E-04 |
| 8077441 | BHLHE40 | basic helix-loop-helix family, member e40 | 2.15 | 5.89E-04 |
| 7920877 | ARHGEF2 | Rho/Rac guanine nucleotide exchange factor (GEF) 2 | 2.14 | 1.57E-04 |
| 8023855 | CYB5A | cytochrome b5 type A (microsomal) | 2.14 | 1.32E-03 |
| 8070341 | BRWD1 | bromodomain and WD repeat domain containing 1 | 2.14 | 1.27E-05 |
| 7915147 | FHL3 | four and a half LIM domains 3 | 2.14 | 1.30E-03 |
| 8132667 | ADCY1 | adenylate cyclase 1 (brain) | 2.14 | 7.11E-04 |
| 7909285 | PFKFB2 | 6-phosphofructo-2-kinase/fructose-2,6-biphosphatase 2 | 2.13 | 2.77E-04 |
| 8040552 | NCOA1 | nuclear receptor coactivator 1 | 2.13 | 1.05E-03 |
| 8070010 | SYNJ1 | synaptojanin 1 | 2.13 | 5.83E-04 |
| 8085360 | TIMP4 | TIMP metallopeptidase inhibitor 4 | 2.12 | 8.34E-04 |
| 8103695 | MFAP3L | microfibrillar-associated protein 3-like | 2.12 | 6.90E-05 |
| 8111915 | SEPP1 | selenoprotein P, plasma, 1 | 2.11 | 9.89E-04 |
| 7982868 | CHAC1 | ChaC, cation transport regulator homolog 1 (E. coli) | 2.11 | 8.74E-04 |
| 7976425 | OTUB2 | OTU domain, ubiquitin aldehyde binding 2 | 2.11 | 1.38E-03 |
| 7984079 | TPM1 | tropomyosin 1 (alpha) | 2.11 | 1.28E-04 |
| 8042310 | SLC1A4 | solute carrier family 1 (glutamate/neutral amino acid transporter), member 4 | 2.11 | 3.75E-05 |
| 8132305 | EEPD1 | endonuclease/exonuclease/phosphatase family domain containing 1 | 2.11 | 3.45E-04 |
| 8059538 | SLC19A3 | solute carrier family 19, member 3 | 2.11 | 1.05E-03 |
| 8091243 | PCOLCE2 | procollagen C-endopeptidase enhancer 2 | 2.10 | 1.30E-03 |
| 8088642 | LRIG1 | leucine-rich repeats and immunoglobulin-like domains 1 | 2.10 | 5.72E-04 |
| 8091698 | SHOX2 | short stature homeobox 2 | 2.10 | 5.02E-04 |
| 8068353 | SLC5A3 | solute carrier family 5 (sodium/myo-inositol cotransporter), member 3 | 2.09 | 7.86E-04 |
| 8068353 | MRPS6 | mitochondrial ribosomal protein S6 | 2.09 | 7.86E-04 |
| 7906469 | DUSP23 | dual specificity phosphatase 23 | 2.09 | 2.22E-05 |
| 8030914 | ZNF610 | zinc finger protein 610 | 2.09 | 2.78E-04 |
| 8030999 | ZNF331 | zinc finger protein 331 | 2.08 | 5.24E-04 |
| 8070129 | DONSON | downstream neighbor of SON | 2.08 | 3.76E-05 |
| 8031640 | ZNF583 | zinc finger protein 583 | 2.08 | 1.12E-04 |
| 7906400 | IFI16 | interferon, gamma-inducible protein 16 | 2.07 | 8.21E-04 |
| 8068902 | RRP1B | ribosomal RNA processing 1 homolog B (S. cerevisiae) | 2.07 | 1.89E-04 |
| 8070557 | ZNF295 | zinc finger protein 295 | 2.06 | 7.13E-05 |
| 7899615 | SERINC2 | serine incorporator 2 | 2.06 | 8.94E-05 |
| 8082075 | DTX3L | deltex 3-like (Drosophila) | 2.05 | 8.22E-04 |
| 8069998 | C21orf59 | chromosome 21 open reading frame 59 | 2.04 | 4.97E-04 |
| 8109563 | NIPAL4 | NIPA-like domain containing 4 | 2.04 | 6.70E-04 |
| 7926037 | PFKFB3 | 6-phosphofructo-2-kinase/fructose-2,6-biphosphatase 3 | 2.04 | 3.12E-04 |
| 8068266 | IFNAR1 | interferon (alpha, beta and omega) receptor 1 | 2.03 | 1.42E-04 |
| 8099982 | APBB2 | amyloid beta (A4) precursor protein-binding, family B, member 2 | 2.03 | 1.83E-04 |
| 8152750 | TMEM65 | transmembrane protein 65 | 2.03 | 6.50E-04 |
| 7908041 | LAMC1 | laminin, gamma 1 (formerly LAMB2) | 2.02 | 7.79E-05 |
| 8108703 | PCDHB6 | protocadherin beta 6 | 2.02 | 3.01E-04 |
| 8172698 | SHROOM4 | shroom family member 4 | 2.02 | 5.84E-04 |
| 8119898 | VEGFA | vascular endothelial growth factor A | 2.02 | 1.27E-07 |
| 8068857 | NDUFV3 | NADH dehydrogenase (ubiquinone) flavoprotein 3, 10kDa | 2.01 | 1.85E-05 |
| 8143341 | JHDM1D | jumonji C domain containing histone demethylase 1 homolog D (S. cerevisiae) | 2.01 | 6.50E-04 |
| 8105067 | PTGER4 | prostaglandin E receptor 4 (subtype EP4) | 2.01 | 7.18E-07 |
| 7902861 | LRRC8B | leucine rich repeat containing 8 family, member B | 2.01 | 3.25E-04 |
| 7918284 | TAF13 | TAF13 RNA polymerase II, TATA box binding protein (TBP)-associated factor, 18kDa | 2.01 | 1.60E-04 |
| 7971296 | EPSTI1 | epithelial stromal interaction 1 (breast) | 2.00 | 8.15E-04 |
| 8092596 | DGKG | diacylglycerol kinase, gamma 90kDa | 2.00 | 7.86E-04 |
| 8039006 | ZNF320 | zinc finger protein 320 | 1.99 | 5.51E-05 |
| 8042283 | HSPC159 | galectin-related protein | 1.99 | 4.29E-04 |
| 8022531 | NPC1 | Niemann-Pick disease, type C1 | 1.99 | 4.39E-04 |
| 8083136 | ATP1B3 | ATPase, Na+/K+ transporting, beta 3 polypeptide | 1.98 | 6.99E-04 |
| 8031857 | ZNF135 | zinc finger protein 135 | 1.97 | 1.17E-03 |
| 8175393 | ARHGEF6 | Rac/Cdc42 guanine nucleotide exchange factor (GEF) 6 | 1.97 | 3.49E-04 |
| 8091432 | COMMD2 | COMM domain containing 2 | 1.97 | 2.12E-04 |
| 8109283 | NDST1 | N-deacetylase/N-sulfotransferase (heparan glucosaminyl) 1 | 1.97 | 3.27E-04 |
| 7929750 | ENTPD7 | ectonucleoside triphosphate diphosphohydrolase 7 | 1.97 | 1.45E-05 |
| 8104035 | SORBS2 | sorbin and SH3 domain containing 2 | 1.97 | 1.80E-04 |
| 7914467 | SPOCD1 | SPOC domain containing 1 | 1.96 | 3.86E-04 |
| 7918026 | EXTL2 | exostoses (multiple)-like 2 | 1.96 | 1.30E-03 |
| 8142424 | GPR85 | G protein-coupled receptor 85 | 1.96 | 5.15E-05 |
| 8083569 | TIPARP | TCDD-inducible poly(ADP-ribose) polymerase | 1.96 | 3.90E-04 |
| 8132465 | HECW1 | HECT, C2 and WW domain containing E3 ubiquitin protein ligase 1 | 1.96 | 1.40E-03 |
| 8114138 | C5orf15 | chromosome 5 open reading frame 15 | 1.95 | 1.16E-03 |
| 8068254 | IL10RB | interleukin 10 receptor, beta | 1.95 | 2.36E-04 |
| 7899029 | MAN1C1 | mannosidase, alpha, class 1C, member 1 | 1.95 | 6.87E-04 |
| 8069753 | CCT8 | chaperonin containing TCP1, subunit 8 (theta) | 1.95 | 7.58E-04 |
| 8107194 | C5orf30 | chromosome 5 open reading frame 30 | 1.95 | 7.14E-04 |
| 8147661 | SPAG1 | sperm associated antigen 1 | 1.94 | 9.63E-04 |
| 8089062 | CLDND1 | claudin domain containing 1 | 1.94 | 8.09E-04 |
| 7903144 | SLC44A3 | solute carrier family 44, member 3 | 1.94 | 2.95E-04 |
| 8085914 | SLC4A7 | solute carrier family 4, sodium bicarbonate cotransporter, member 7 | 1.93 | 2.83E-04 |
| 7966690 | TBX3 | T-box 3 | 1.93 | 1.63E-04 |
| 7917503 | GBP3 | guanylate binding protein 3 | 1.93 | 4.81E-05 |
| 8116910 | HIVEP1 | human immunodeficiency virus type I enhancer binding protein 1 | 1.92 | 4.50E-04 |
| 8068375 | FAM165B | family with sequence similarity 165, member B | 1.92 | 1.06E-03 |
| 8086372 | ULK4 | unc-51-like kinase 4 (C. elegans) | 1.92 | 6.88E-04 |
| 8089954 | IQCB1 | IQ motif containing B1 | 1.91 | 4.38E-05 |
| 7989501 | CA12 | carbonic anhydrase XII | 1.91 | 7.98E-04 |
| 8037374 | PLAUR | plasminogen activator, urokinase receptor | 1.91 | 6.82E-06 |
| 8025478 | ZNF559 | zinc finger protein 559 | 1.90 | 2.90E-05 |
| 8152828 | GSDMC | gasdermin C | 1.90 | 9.42E-04 |
| 8083901 | FNDC3B | fibronectin type III domain containing 3B | 1.90 | 1.05E-04 |
| 8130211 | SYNE1 | spectrin repeat containing, nuclear envelope 1 | 1.90 | 2.28E-04 |
| 8142407 | TMEM168 | transmembrane protein 168 | 1.90 | 7.68E-04 |
| 7965094 | E2F7 | E2F transcription factor 7 | 1.89 | 1.73E-04 |
| 7977786 | SLC7A7 | solute carrier family 7 (cationic amino acid transporter, y+ system), member 7 | 1.89 | 1.05E-03 |
| 8070912 | SLC19A1 | solute carrier family 19 (folate transporter), member 1 | 1.89 | 2.80E-04 |
| 8141076 | PON2 | paraoxonase 2 | 1.89 | 1.41E-03 |
| 8042211 | B3GNT2 | UDP-GlcNAc:betaGal beta-1,3-N-acetylglucosaminyltransferase 2 | 1.89 | 1.29E-03 |
| 8044258 | LIMS1 | LIM and senescent cell antigen-like domains 1 | 1.88 | 1.11E-03 |
| 7900365 | MFSD2A | major facilitator superfamily domain containing 2A | 1.88 | 5.66E-06 |
| 8167973 | HEPH | hephaestin | 1.88 | 1.00E-03 |
| 8146579 | CHD7 | chromodomain helicase DNA binding protein 7 | 1.87 | 1.41E-04 |
| 8068974 | TRAPPC10 | trafficking protein particle complex 10 | 1.87 | 5.03E-04 |
| 8137352 | SLC4A2 | solute carrier family 4, anion exchanger, member 2 (erythrocyte membrane protein band 3-like 1) | 1.87 | 9.32E-04 |
| 7962579 | AMIGO2 | adhesion molecule with Ig-like domain 2 | 1.86 | 2.26E-04 |
| 8031714 | ZNF460 | zinc finger protein 460 | 1.86 | 4.50E-04 |
| 8004184 | XAF1 | XIAP associated factor 1 | 1.85 | 6.45E-04 |
| 8030997 | ZNF813 | zinc finger protein 813 | 1.84 | 6.81E-04 |
| 7922756 | NMNAT2 | nicotinamide nucleotide adenylyltransferase 2 | 1.84 | 1.38E-03 |
| 7909225 | DYRK3 | dual-specificity tyrosine-(Y)-phosphorylation regulated kinase 3 | 1.84 | 7.28E-04 |
| 8093053 | TFRC | transferrin receptor (p90, CD71) | 1.84 | 1.07E-05 |
| 8029688 | CD3EAP | CD3e molecule, epsilon associated protein | 1.83 | 1.06E-03 |
| 8123951 | C6orf105 | chromosome 6 open reading frame 105 | 1.83 | 7.46E-05 |
| 7924476 | TAF1A | TATA box binding protein (TBP)-associated factor, RNA polymerase I, A, 48kDa | 1.83 | 8.21E-04 |
| 8093130 | RNF168 | ring finger protein 168 | 1.83 | 9.59E-04 |
| 8029437 | PVR | poliovirus receptor | 1.83 | 4.15E-04 |
| 8141522 | C7orf61 | chromosome 7 open reading frame 61 | 1.83 | 6.37E-04 |
| 8120783 | MYO6 | myosin VI | 1.82 | 3.47E-04 |
| 7954559 | PPFIBP1 | PTPRF interacting protein, binding protein 1 (liprin beta 1) | 1.82 | 5.56E-04 |
| 8139656 | GRB10 | growth factor receptor-bound protein 10 | 1.82 | 9.78E-04 |
| 8105348 | GPX8 | glutathione peroxidase 8 (putative) | 1.82 | 1.19E-03 |
| 8070330 | PSMG1 | proteasome (prosome, macropain) assembly chaperone 1 | 1.81 | 1.85E-04 |
| 7960850 | SLC2A14 | solute carrier family 2 (facilitated glucose transporter), member 14 | 1.81 | 7.32E-04 |
| 7907370 | DNM3 | dynamin 3 | 1.81 | 1.30E-03 |
| 8081115 | ARL13B | ADP-ribosylation factor-like 13B | 1.81 | 5.80E-04 |
| 8070102 | GART | phosphoribosylglycinamide formyltransferase, phosphoribosylglycinamide synthetase, phosphoribosylaminoimidazole synthetase | 1.81 | 1.16E-04 |
| 7957260 | GLIPR1 | GLI pathogenesis-related 1 | 1.81 | 3.91E-05 |
| 8028200 | ZNF567 | zinc finger protein 567 | 1.80 | 1.39E-03 |
| 8151816 | GEM | GTP binding protein overexpressed in skeletal muscle | 1.80 | 6.49E-04 |
| 8089820 | GPR156 | G protein-coupled receptor 156 | 1.80 | 1.37E-03 |
| 8068522 | TTC3 | tetratricopeptide repeat domain 3 | 1.80 | 6.22E-04 |
| 8085788 | NKIRAS1 | NFKB inhibitor interacting Ras-like 1 | 1.79 | 1.10E-03 |
| 7923812 | RAB7L1 | RAB7, member RAS oncogene family-like 1 | 1.79 | 9.56E-04 |
| 8091048 | COPB2 | coatomer protein complex, subunit beta 2 (beta prime) | 1.78 | 1.29E-03 |
| 8084423 | PSMD2 | proteasome (prosome, macropain) 26S subunit, non-ATPase, 2 | 1.78 | 1.25E-03 |
| 8095021 | SPATA18 | spermatogenesis associated 18 homolog (rat) | 1.78 | 7.95E-04 |
| 8081676 | GTPBP8 | GTP-binding protein 8 (putative) | 1.78 | 7.64E-04 |
| 8086572 | FYCO1 | FYVE and coiled-coil domain containing 1 | 1.78 | 7.32E-04 |
| 8112865 | SERINC5 | serine incorporator 5 | 1.77 | 3.75E-06 |
| 8039706 | ZNF256 | zinc finger protein 256 | 1.77 | 1.01E-03 |
| 8091648 | SSR3 | signal sequence receptor, gamma (translocon-associated protein gamma) | 1.77 | 6.16E-04 |
| 7970949 | MAB21L1 | mab-21-like 1 (C. elegans) | 1.77 | 3.77E-05 |
| 8070269 | DSCR3 | Down syndrome critical region gene 3 | 1.77 | 1.17E-04 |
| 8090577 | MBD4 | methyl-CpG binding domain protein 4 | 1.77 | 1.10E-03 |
| 7964602 | LRIG3 | leucine-rich repeats and immunoglobulin-like domains 3 | 1.76 | 4.40E-04 |
| 8060344 | TRIB3 | tribbles homolog 3 (Drosophila) | 1.76 | 7.86E-04 |
| 8065344 | FOXA2 | forkhead box A2 | 1.76 | 1.46E-04 |
| 8123864 | TFAP2A | transcription factor AP-2 alpha (activating enhancer binding protein 2 alpha) | 1.76 | 8.35E-04 |
| 8168817 | DRP2 | dystrophin related protein 2 | 1.75 | 5.17E-04 |
| 8078138 | EAF1 | ELL associated factor 1 | 1.75 | 2.78E-04 |
| 7916609 | JUN | jun proto-oncogene | 1.75 | 1.42E-03 |
| 7958410 | FICD | FIC domain containing | 1.75 | 8.01E-04 |
| 8131631 | HDAC9 | histone deacetylase 9 | 1.75 | 4.65E-05 |
| 8025992 | ZNF788 | zinc finger family member 788 | 1.75 | 7.13E-04 |
| 7922823 | EDEM3 | ER degradation enhancer, mannosidase alpha-like 3 | 1.75 | 1.61E-04 |
| 8036351 | ZNF850 | zinc finger protein 850 | 1.74 | 4.27E-04 |
| 8069644 | APP | amyloid beta (A4) precursor protein | 1.74 | 7.63E-04 |
| 7904433 | PHGDH | phosphoglycerate dehydrogenase | 1.74 | 1.25E-03 |
| 8141150 | ASNS | asparagine synthetase (glutamine-hydrolyzing) | 1.74 | 1.07E-04 |
| 8068460 | MORC3 | MORC family CW-type zinc finger 3 | 1.74 | 6.42E-05 |
| 8089647 | KIAA2018 | KIAA2018 | 1.73 | 1.36E-03 |
| 8140534 | SEMA3C | sema domain, immunoglobulin domain (Ig), short basic domain, secreted, (semaphorin) 3C | 1.73 | 1.08E-03 |
| 8157383 | COL27A1 | collagen, type XXVII, alpha 1 | 1.73 | 1.39E-03 |
| 7913558 | LUZP1 | leucine zipper protein 1 | 1.73 | 4.22E-04 |
| 7947462 | ABTB2 | ankyrin repeat and BTB (POZ) domain containing 2 | 1.73 | 4.63E-04 |
| 8031784 | ZNF211 | zinc finger protein 211 | 1.73 | 6.73E-04 |
| 8031784 | ZNF134 | zinc finger protein 134 | 1.73 | 6.73E-04 |
| 8114396 | CDC23 | cell division cycle 23 homolog (S. cerevisiae) | 1.73 | 9.73E-04 |
| 8060897 | PLCB4 | phospholipase C, beta 4 | 1.72 | 4.34E-04 |
| 7896759 | LOC643837 | hypothetical LOC643837 | 1.72 | 2.28E-04 |
| 8115147 | CD74 | CD74 molecule, major histocompatibility complex, class II invariant chain | 1.72 | 2.69E-04 |
| 8142194 | LAMB1 | laminin, beta 1 | 1.72 | 7.53E-04 |
| 8078386 | GPD1L | glycerol-3-phosphate dehydrogenase 1-like | 1.72 | 1.23E-03 |
| 8160274 | MGC24103 | hypothetical MGC24103 | 1.72 | 4.57E-05 |
| 8038998 | ZNF468 | zinc finger protein 468 | 1.72 | 6.56E-05 |
| 8068551 | DYRK1A | dual-specificity tyrosine-(Y)-phosphorylation regulated kinase 1A | 1.71 | 3.07E-04 |
| 8068612 | WRB | tryptophan rich basic protein | 1.71 | 5.84E-04 |
| 8082066 | FAM162A | family with sequence similarity 162, member A | 1.71 | 1.17E-03 |
| 7972713 | EFNB2 | ephrin-B2 | 1.71 | 4.85E-04 |
| 8072710 | APOL6 | apolipoprotein L, 6 | 1.70 | 2.20E-04 |
| 8038967 | ZNF83 | zinc finger protein 83 | 1.70 | 4.60E-04 |
| 7962274 | KIF21A | kinesin family member 21A | 1.70 | 5.27E-04 |
| 8115681 | PANK3 | pantothenate kinase 3 | 1.70 | 3.18E-04 |
| 8083360 | MED12L | mediator complex subunit 12-like | 1.70 | 5.84E-04 |
| 8135436 | SLC26A4 | solute carrier family 26, member 4 | 1.70 | 1.22E-03 |
| 8180363 | MPRIP | myosin phosphatase Rho interacting protein | 1.70 | 1.15E-03 |
| 8130176 | ULBP3 | UL16 binding protein 3 | 1.70 | 5.20E-04 |
| 7901969 | ROR1 | receptor tyrosine kinase-like orphan receptor 1 | 1.70 | 1.08E-03 |
| 8180362 | MPRIP | myosin phosphatase Rho interacting protein | 1.70 | 1.28E-03 |
| 8068062 | USP16 | ubiquitin specific peptidase 16 | 1.69 | 1.28E-05 |
| 8081235 | COL8A1 | collagen, type VIII, alpha 1 | 1.69 | 1.37E-03 |
| 8143327 | PARP12 | poly (ADP-ribose) polymerase family, member 12 | 1.68 | 1.49E-04 |
| 8093852 | MSX1 | msh homeobox 1 | 1.68 | 1.26E-03 |
| 7925028 | FAM89A | family with sequence similarity 89, member A | 1.68 | 4.23E-04 |
| 8085999 | CMTM6 | CKLF-like MARVEL transmembrane domain containing 6 | 1.67 | 1.13E-03 |
| 8068289 | SON | SON DNA binding protein | 1.67 | 3.36E-04 |
| 8104788 | RAI14 | retinoic acid induced 14 | 1.67 | 6.56E-04 |
| 7953469 | GNB3 | guanine nucleotide binding protein (G protein), beta polypeptide 3 | 1.67 | 2.57E-04 |
| 8068363 | C21orf82 | chromosome 21 open reading frame 82 | 1.66 | 6.28E-04 |
| 8044499 | SLC20A1 | solute carrier family 20 (phosphate transporter), member 1 | 1.66 | 5.15E-04 |
| 8054712 | IL1A | interleukin 1, alpha | 1.66 | 4.24E-04 |
| 8080419 | GNL3 | guanine nucleotide binding protein-like 3 (nucleolar) | 1.66 | 1.16E-03 |
| 8176191 | HMGN1 | high mobility group nucleosome binding domain 1 | 1.66 | 1.29E-03 |
| 8081620 | TAGLN3 | transgelin 3 | 1.66 | 4.97E-04 |
| 8062134 | GDF5OS | growth differentiation factor 5 opposite strand | 1.65 | 8.52E-04 |
| 8154656 | DMRTA1 | DMRT-like family A1 | 1.65 | 3.70E-04 |
| 8069711 | LTN1 | listerin E3 ubiquitin protein ligase 1 | 1.65 | 9.94E-05 |
| 8138718 | HOXA2 | homeobox A2 | 1.65 | 9.26E-04 |
| 8110392 | TMED9 | transmembrane emp24 protein transport domain containing 9 | 1.65 | 6.63E-04 |
| 7924701 | ACBD3 | acyl-CoA binding domain containing 3 | 1.64 | 9.95E-04 |
| 7902158 | TCTEX1D1 | Tctex1 domain containing 1 | 1.64 | 1.41E-03 |
| 8079021 | CTNNB1 | catenin (cadherin-associated protein), beta 1, 88kDa | 1.64 | 1.17E-03 |
| 8013622 | SGK494 | uncharacterized serine/threonine-protein kinase SgK494 | 1.64 | 4.46E-05 |
| 8069620 | MRPL39 | mitochondrial ribosomal protein L39 | 1.64 | 3.47E-04 |
| 8143367 | SLC37A3 | solute carrier family 37 (glycerol-3-phosphate transporter), member 3 | 1.64 | 7.65E-04 |
| 7902476 | FAM73A | family with sequence similarity 73, member A | 1.64 | 1.01E-03 |
| 7915015 | GNL2 | guanine nucleotide binding protein-like 2 (nucleolar) | 1.64 | 4.64E-04 |
| 8092201 | TBL1XR1 | transducin (beta)-like 1 X-linked receptor 1 | 1.63 | 7.17E-04 |
| 8107909 | SLC22A4 | solute carrier family 22 (organic cation/ergothioneine transporter), member 4 | 1.63 | 4.58E-04 |
| 8152280 | LRP12 | low density lipoprotein receptor-related protein 12 | 1.63 | 9.51E-04 |
| 8070799 | UBE2G2 | ubiquitin-conjugating enzyme E2G 2 (UBC7 homolog, yeast) | 1.62 | 5.91E-05 |
| 8068105 | BACH1 | BTB and CNC homology 1, basic leucine zipper transcription factor 1 | 1.62 | 1.34E-03 |
| 8105504 | LOC728153 | similar to FAM133B protein | 1.62 | 1.34E-03 |
| 8105504 | FAM133B | family with sequence similarity 133, member B | 1.62 | 1.34E-03 |
| 8049532 | LRRFIP1 | leucine rich repeat (in FLII) interacting protein 1 | 1.62 | 1.24E-03 |
| 8138834 | FKBP14 | FK506 binding protein 14, 22 kDa | 1.61 | 8.99E-04 |
| 8022488 | ABHD3 | abhydrolase domain containing 3 | 1.61 | 6.18E-04 |
| 8080773 | RPP14 | ribonuclease P/MRP 14kDa subunit | 1.61 | 8.79E-04 |
| 8146687 | ADHFE1 | alcohol dehydrogenase, iron containing, 1 | 1.61 | 8.65E-04 |
| 8172043 | SRPX | sushi-repeat containing protein, X-linked | 1.60 | 1.33E-03 |
| 7933084 | NAMPT | nicotinamide phosphoribosyltransferase | 1.60 | 5.66E-05 |
| 8083677 | SCHIP1 | schwannomin interacting protein 1 | 1.60 | 1.23E-03 |
| 8028227 | ZNF383 | zinc finger protein 383 | 1.60 | 8.69E-04 |
| 8028389 | SPRED3 | sprouty-related, EVH1 domain containing 3 | 1.59 | 1.31E-03 |
| 8036324 | ZNF260 | zinc finger protein 260 | 1.59 | 1.32E-03 |
| 8142120 | NAMPT | nicotinamide phosphoribosyltransferase | 1.59 | 4.32E-04 |
| 8091799 | C3orf57 | chromosome 3 open reading frame 57 | 1.59 | 1.97E-04 |
| 8070160 | ATP5O | ATP synthase, H+ transporting, mitochondrial F1 complex, O subunit | 1.59 | 8.37E-04 |
| 8088092 | RFT1 | RFT1 homolog (S. cerevisiae) | 1.59 | 5.22E-04 |
| 7909441 | G0S2 | G0/G1switch 2 | 1.59 | 2.10E-04 |
| 8023735 | TMX3 | thioredoxin-related transmembrane protein 3 | 1.59 | 7.58E-04 |
| 8138735 | HOXA5 | homeobox A5 | 1.58 | 7.76E-04 |
| 7921110 | ISG20L2 | interferon stimulated exonuclease gene 20kDa-like 2 | 1.58 | 1.05E-03 |
| 8025984 | ZNF844 | zinc finger protein 844 | 1.58 | 8.18E-04 |
| 8104680 | C5orf22 | chromosome 5 open reading frame 22 | 1.57 | 1.38E-03 |
| 7917576 | GBP5 | guanylate binding protein 5 | 1.57 | 1.28E-03 |
| 8078529 | STAC | SH3 and cysteine rich domain | 1.57 | 1.36E-03 |
| 7974851 | HIF1A | hypoxia inducible factor 1, alpha subunit (basic helix-loop-helix transcription factor) | 1.57 | 7.17E-04 |
| 8031762 | ZNF549 | zinc finger protein 549 | 1.57 | 6.12E-04 |
| 8133788 | PTPN12 | protein tyrosine phosphatase, non-receptor type 12 | 1.57 | 4.17E-05 |
| 7976012 | NRXN3 | neurexin 3 | 1.57 | 6.58E-04 |
| 8069676 | ADAMTS1 | ADAM metallopeptidase with thrombospondin type 1 motif, 1 | 1.57 | 1.30E-03 |
| 8102482 | SEC24D | SEC24 family, member D (S. cerevisiae) | 1.56 | 1.14E-03 |
| 7920852 | KIAA0907 | KIAA0907 | 1.56 | 7.96E-04 |
| 8084947 | FBXO45 | F-box protein 45 | 1.56 | 4.41E-05 |
| 8038913 | ZNF649 | zinc finger protein 649 | 1.56 | 9.18E-04 |
| 8081158 | ARL6 | ADP-ribosylation factor-like 6 | 1.56 | 6.82E-04 |
| 7907171 | BLZF1 | basic leucine zipper nuclear factor 1 | 1.56 | 1.22E-03 |
| 8081171 | CRYBG3 | beta-gamma crystallin domain containing 3 | 1.56 | 9.77E-04 |
| 8127841 | PGM3 | phosphoglucomutase 3 | 1.55 | 5.12E-04 |
| 7915286 | PPT1 | palmitoyl-protein thioesterase 1 | 1.55 | 9.75E-04 |
| 7951036 | TAF1D | TATA box binding protein (TBP)-associated factor, RNA polymerase I, D, 41kDa | 1.55 | 2.89E-04 |
| 7951036 | SNORD5 | small nucleolar RNA, C/D box 5 | 1.55 | 2.89E-04 |
| 8177955 | MICB | MHC class I polypeptide-related sequence B | 1.55 | 1.21E-03 |
| 8092905 | LSG1 | large subunit GTPase 1 homolog (S. cerevisiae) | 1.55 | 8.97E-04 |
| 8136115 | FAM40B | family with sequence similarity 40, member B | 1.55 | 3.98E-04 |
| 8021603 | SERPINB13 | serpin peptidase inhibitor, clade B (ovalbumin), member 13 | 1.54 | 8.59E-04 |
| 8092073 | EIF5A2 | eukaryotic translation initiation factor 5A2 | 1.54 | 5.16E-04 |
| 7948229 | SLC43A3 | solute carrier family 43, member 3 | 1.54 | 2.59E-04 |
| 8036395 | ZNF569 | zinc finger protein 569 | 1.54 | 4.65E-04 |
| 8144036 | XRCC2 | X-ray repair complementing defective repair in Chinese hamster cells 2 | 1.54 | 4.96E-05 |
| 8104234 | TRIP13 | thyroid hormone receptor interactor 13 | 1.54 | 3.80E-04 |
| 8077731 | FANCD2 | Fanconi anemia, complementation group D2 | 1.53 | 1.01E-04 |
| 8038954 | ZNF616 | zinc finger protein 616 | 1.52 | 4.38E-04 |
| 7924119 | INTS7 | integrator complex subunit 7 | 1.52 | 9.66E-04 |
| 8113073 | ARRDC3 | arrestin domain containing 3 | 1.52 | 3.19E-05 |
| 8086985 | COL7A1 | collagen, type VII, alpha 1 | 1.52 | 4.07E-04 |
| 8108238 | SMAD5 | SMAD family member 5 | 1.51 | 1.38E-03 |
| 8096635 | NFKB1 | nuclear factor of kappa light polypeptide gene enhancer in B-cells 1 | 1.51 | 9.97E-04 |
| 8133914 | DMTF1 | cyclin D binding myb-like transcription factor 1 | 1.51 | 1.32E-03 |
| 8111629 | NUP155 | nucleoporin 155kDa | 1.51 | 6.98E-04 |
| 8078360 | STT3B | STT3, subunit of the oligosaccharyltransferase complex, homolog B (S. cerevisiae) | 1.50 | 6.03E-04 |
| 8142143 | COG5 | component of oligomeric golgi complex 5 | 1.50 | 7.09E-05 |
| 8085660 | DPH3 | DPH3, KTI11 homolog (S. cerevisiae) | 1.50 | 3.64E-04 |
| 8113250 | ERAP1 | endoplasmic reticulum aminopeptidase 1 | 1.50 | 4.41E-04 |
| 8118594 | HLA-DPB1 | major histocompatibility complex, class II, DP beta 1 | 1.49 | 8.43E-04 |
| 8133818 | PHTF2 | putative homeodomain transcription factor 2 | 1.49 | 2.52E-04 |
| 8106393 | F2R | coagulation factor II (thrombin) receptor | 1.49 | 8.30E-05 |
| 7940153 | FAM111A | family with sequence similarity 111, member A | 1.48 | 1.12E-04 |
| 8090448 | RUVBL1 | RuvB-like 1 (E. coli) | 1.47 | 1.41E-03 |
| 8133030 | GABPA | GA binding protein transcription factor, alpha subunit 60kDa | 1.47 | 3.02E-05 |
| 8151756 | TMEM55A | transmembrane protein 55A | 1.47 | 8.53E-04 |
| 8023561 | LMAN1 | lectin, mannose-binding, 1 | 1.47 | 1.07E-03 |
| 8031778 | ZNF530 | zinc finger protein 530 | 1.47 | 1.37E-03 |
| 7918847 | SIKE1 | suppressor of IKBKE 1 | 1.46 | 8.82E-04 |
| 8178059 | LY6G5B | lymphocyte antigen 6 complex, locus G5B | 1.46 | 1.37E-03 |
| 7925257 | LYST | lysosomal trafficking regulator | 1.46 | 2.66E-04 |
| 7904364 | WDR3 | WD repeat domain 3 | 1.46 | 2.31E-04 |
| 8140864 | CYP51A1 | cytochrome P450, family 51, subfamily A, polypeptide 1 | 1.46 | 9.67E-04 |
| 8107356 | DCP2 | DCP2 decapping enzyme homolog (S. cerevisiae) | 1.46 | 3.73E-04 |
| 8088700 | TMF1 | TATA element modulatory factor 1 | 1.46 | 6.43E-04 |
| 8035177 | SLC35E1 | solute carrier family 35, member E1 | 1.46 | 7.28E-04 |
| 8135323 | RINT1 | RAD50 interactor 1 | 1.46 | 6.78E-04 |
| 8083282 | HPS3 | Hermansky-Pudlak syndrome 3 | 1.46 | 1.03E-03 |
| 8141107 | SLC25A13 | solute carrier family 25, member 13 (citrin) | 1.46 | 1.56E-04 |
| 8136067 | TSPAN33 | tetraspanin 33 | 1.45 | 8.65E-04 |
| 8106727 | FLJ41309 | hypothetical LOC645079 | 1.45 | 9.52E-04 |
| 8106727 | ATP6AP1L | ATPase, H+ transporting, lysosomal accessory protein 1-like | 1.45 | 9.52E-04 |
| 8028241 | ZNF527 | zinc finger protein 527 | 1.45 | 6.71E-04 |
| 8009277 | RGS9 | regulator of G-protein signaling 9 | 1.44 | 5.71E-04 |
| 7965040 | PHLDA1 | pleckstrin homology-like domain, family A, member 1 | 1.44 | 9.53E-04 |
| 8083183 | U2SURP | U2 snRNP-associated SURP domain containing | 1.44 | 5.41E-05 |
| 8081316 | TFG | TRK-fused gene | 1.44 | 1.07E-03 |
| 8083757 | NMD3 | NMD3 homolog (S. cerevisiae) | 1.44 | 4.43E-05 |
| 8134789 | PILRB | paired immunoglobin-like type 2 receptor beta | 1.43 | 8.92E-04 |
| 8107868 | CDC42SE2 | CDC42 small effector 2 | 1.43 | 1.21E-03 |
| 8086494 | ZNF852 | zinc finger protein 852 | 1.43 | 1.10E-03 |
| 8070257 | PIGP | phosphatidylinositol glycan anchor biosynthesis, class P | 1.43 | 9.74E-04 |
| 8027674 | ZNF302 | zinc finger protein 302 | 1.43 | 1.16E-03 |
| 8107578 | SRFBP1 | serum response factor binding protein 1 | 1.43 | 1.86E-04 |
| 8089988 | CCDC58 | coiled-coil domain containing 58 | 1.43 | 3.88E-04 |
| 7925622 | AHCTF1 | AT hook containing transcription factor 1 | 1.42 | 5.73E-04 |
| 8030946 | ZNF808 | zinc finger protein 808 | 1.42 | 7.34E-05 |
| 8091009 | PIK3CB | phosphoinositide-3-kinase, catalytic, beta polypeptide | 1.42 | 1.24E-03 |
| 7899829 | S100PBP | S100P binding protein | 1.42 | 1.27E-03 |
| 8088001 | NEK4 | NIMA (never in mitosis gene a)-related kinase 4 | 1.42 | 6.32E-04 |
| 8091190 | ATR | ataxia telangiectasia and Rad3 related | 1.41 | 3.02E-04 |
| 7916969 | ZRANB2 | zinc finger, RAN-binding domain containing 2 | 1.41 | 7.83E-04 |
| 8140151 | RFC2 | replication factor C (activator 1) 2, 40kDa | 1.41 | 2.05E-04 |
| 8012896 | PMP22 | peripheral myelin protein 22 | 1.41 | 5.57E-04 |
| 8112312 | DIMT1L | DIM1 dimethyladenosine transferase 1-like (S. cerevisiae) | 1.41 | 8.53E-04 |
| 8142061 | PUS7 | pseudouridylate synthase 7 homolog (S. cerevisiae) | 1.41 | 9.27E-04 |
| 8051998 | MCFD2 | multiple coagulation factor deficiency 2 | 1.41 | 5.12E-04 |
| 8109802 | RARS | arginyl-tRNA synthetase | 1.41 | 1.19E-03 |
| 8003298 | SLC7A5 | solute carrier family 7 (cationic amino acid transporter, y+ system), member 5 | 1.40 | 5.87E-04 |
| 8084955 | PIGX | phosphatidylinositol glycan anchor biosynthesis, class X | 1.40 | 5.68E-04 |
| 7900216 | AKIRIN1 | akirin 1 | 1.39 | 4.49E-04 |
| 8134122 | AKAP9 | A kinase (PRKA) anchor protein (yotiao) 9 | 1.39 | 8.09E-04 |
| 8140398 | YWHAG | tyrosine 3-monooxygenase/tryptophan 5-monooxygenase activation protein, gamma polypeptide | 1.39 | 3.71E-04 |
| 8054254 | AFF3 | AF4/FMR2 family, member 3 | 1.39 | 7.43E-04 |
| 8088339 | ARF4 | ADP-ribosylation factor 4 | 1.38 | 5.28E-04 |
| 8056220 | AHCTF1 | AT hook containing transcription factor 1 | 1.38 | 4.02E-05 |
| 8131479 | MIOS | missing oocyte, meiosis regulator, homolog (Drosophila) | 1.38 | 4.29E-04 |
| 8025945 | ZNF441 | zinc finger protein 441 | 1.38 | 1.88E-04 |
| 8084045 | MFN1 | mitofusin 1 | 1.38 | 1.13E-03 |
| 8059177 | TUBA4A | tubulin, alpha 4a | 1.38 | 2.71E-05 |
| 7919193 | NUDT4P1 | nudix (nucleoside diphosphate linked moiety X)-type motif 4 pseudogene 1 | 1.38 | 3.62E-04 |
| 7919193 | NUDT4 | nudix (nucleoside diphosphate linked moiety X)-type motif 4 | 1.38 | 3.62E-04 |
| 8136983 | OR2A9P | olfactory receptor, family 2, subfamily A, member 9 pseudogene | 1.38 | 1.03E-03 |
| 8136983 | OR2A42 | olfactory receptor, family 2, subfamily A, member 42 | 1.38 | 1.03E-03 |
| 8136983 | OR2A20P | olfactory receptor, family 2, subfamily A, member 20 pseudogene | 1.38 | 1.03E-03 |
| 8136983 | OR2A1 | olfactory receptor, family 2, subfamily A, member 1 | 1.38 | 1.03E-03 |
| 8105181 | MRPS30 | mitochondrial ribosomal protein S30 | 1.37 | 1.59E-04 |
| 8038981 | ZNF611 | zinc finger protein 611 | 1.37 | 1.05E-03 |
| 7921713 | TSTD1 | thiosulfate sulfurtransferase (rhodanese)-like domain containing 1 | 1.37 | 1.24E-05 |
| 7921713 | F11R | F11 receptor | 1.37 | 1.24E-05 |
| 7947338 | PAX6 | paired box 6 | 1.37 | 1.23E-03 |
| 8121895 | TRMT11 | tRNA methyltransferase 11 homolog (S. cerevisiae) | 1.36 | 9.09E-04 |
| 8136177 | KLHDC10 | kelch domain containing 10 | 1.36 | 9.06E-04 |
| 7923659 | PPP1R15B | protein phosphatase 1, regulatory (inhibitor) subunit 15B | 1.36 | 4.88E-05 |
| 8048864 | CCL20 | chemokine (C-C motif) ligand 20 | 1.36 | 9.34E-04 |
| 8089040 | MINA | MYC induced nuclear antigen | 1.35 | 7.02E-04 |
| 8111960 | C5orf34 | chromosome 5 open reading frame 34 | 1.35 | 5.30E-04 |
| 8143028 | CHCHD3 | coiled-coil-helix-coiled-coil-helix domain containing 3 | 1.35 | 1.34E-03 |
| 8086077 | CLASP2 | cytoplasmic linker associated protein 2 | 1.35 | 4.84E-04 |
| 8168107 | AWAT1 | acyl-CoA wax alcohol acyltransferase 1 | 1.35 | 7.89E-05 |
| 8081612 | ABHD10 | abhydrolase domain containing 10 | 1.35 | 8.48E-04 |
| 8075971 | ANKRD54 | ankyrin repeat domain 54 | 1.34 | 1.17E-03 |
| 8135955 | CALU | calumenin | 1.34 | 1.15E-03 |
| 7925364 | HEATR1 | HEAT repeat containing 1 | 1.34 | 2.36E-04 |
| 8133610 | STAG3L3 | stromal antigen 3-like 3 | 1.34 | 1.31E-04 |
| 8133610 | STAG3L2 | stromal antigen 3-like 2 | 1.34 | 1.31E-04 |
| 8133610 | STAG3L1 | stromal antigen 3-like 1 | 1.34 | 1.31E-04 |
| 8140196 | STAG3L3 | stromal antigen 3-like 3 | 1.33 | 2.20E-04 |
| 8140196 | STAG3L2 | stromal antigen 3-like 2 | 1.33 | 2.20E-04 |
| 8140196 | STAG3L1 | stromal antigen 3-like 1 | 1.33 | 2.20E-04 |
| 8180268 | CYP51A1 | cytochrome P450, family 51, subfamily A, polypeptide 1 | 1.33 | 1.41E-04 |
| 8144153 | NCAPG2 | non-SMC condensin II complex, subunit G2 | 1.33 | 1.41E-03 |
| 8096899 | C4orf32 | chromosome 4 open reading frame 32 | 1.33 | 1.14E-03 |
| 8090678 | MRPL3 | mitochondrial ribosomal protein L3 | 1.33 | 1.20E-03 |
| 8164883 | SURF4 | surfeit 4 | 1.32 | 1.21E-03 |
| 8139977 | STAG3L3 | stromal antigen 3-like 3 | 1.32 | 1.70E-04 |
| 8139977 | STAG3L2 | stromal antigen 3-like 2 | 1.32 | 1.70E-04 |
| 8139977 | STAG3L1 | stromal antigen 3-like 1 | 1.32 | 1.70E-04 |
| 8142774 | RBM28 | RNA binding motif protein 28 | 1.32 | 3.38E-05 |
| 8136401 | NUP205 | nucleoporin 205kDa | 1.32 | 5.50E-04 |
| 8042381 | PNO1 | partner of NOB1 homolog (S. cerevisiae) | 1.32 | 8.22E-04 |
| 7957433 | LRRIQ1 | leucine-rich repeats and IQ motif containing 1 | 1.31 | 2.73E-04 |
| 7965681 | IKBIP | IKBKB interacting protein | 1.31 | 1.18E-03 |
| 8085852 | NGLY1 | N-glycanase 1 | 1.31 | 8.56E-04 |
| 7912224 | SLC2A5 | solute carrier family 2 (facilitated glucose/fructose transporter), member 5 | 1.31 | 6.57E-04 |
| 8143629 | OR2A9P | olfactory receptor, family 2, subfamily A, member 9 pseudogene | 1.31 | 1.28E-03 |
| 8143629 | OR2A42 | olfactory receptor, family 2, subfamily A, member 42 | 1.31 | 1.28E-03 |
| 8143629 | OR2A20P | olfactory receptor, family 2, subfamily A, member 20 pseudogene | 1.31 | 1.28E-03 |
| 8143629 | OR2A1 | olfactory receptor, family 2, subfamily A, member 1 | 1.31 | 1.28E-03 |
| 7915926 | STIL | SCL/TAL1 interrupting locus | 1.31 | 5.91E-04 |
| 8090772 | TOPBP1 | topoisomerase (DNA) II binding protein 1 | 1.30 | 8.22E-04 |
| 8140955 | CDK6 | cyclin-dependent kinase 6 | 1.30 | 1.34E-03 |
| 8033789 | ZNF121 | zinc finger protein 121 | 1.29 | 4.14E-04 |
| 8139033 | AOAH | acyloxyacyl hydrolase (neutrophil) | 1.29 | 1.38E-04 |
| 7942832 | C11orf82 | chromosome 11 open reading frame 82 | 1.29 | 9.54E-04 |
| 8135688 | NAA38 | N(alpha)-acetyltransferase 38, NatC auxiliary subunit | 1.29 | 4.39E-04 |
| 8063634 | MGC4294 | hypothetical MGC4294 | 1.28 | 2.44E-04 |
| 8145977 | PLEKHA2 | pleckstrin homology domain containing, family A (phosphoinositide binding specific) member 2 | 1.28 | 1.10E-03 |
| 8131614 | AHR | aryl hydrocarbon receptor | 1.28 | 1.38E-03 |
| 8081241 | C3orf26 | chromosome 3 open reading frame 26 | 1.28 | 1.02E-03 |
| 8126371 | CCND3 | cyclin D3 | 1.27 | 1.21E-03 |
| 8134613 | ZNF789 | zinc finger protein 789 | 1.27 | 1.03E-04 |
| 8169459 | SNORA35 | small nucleolar RNA, H/ACA box 35 | 1.26 | 1.26E-03 |
| 8145122 | SLC39A14 | solute carrier family 39 (zinc transporter), member 14 | 1.26 | 1.29E-04 |
| 8107282 | WDR36 | WD repeat domain 36 | 1.25 | 1.04E-04 |
| 8026982 | MPV17L2 | MPV17 mitochondrial membrane protein-like 2 | 1.25 | 6.32E-05 |
| 7916316 | TMEM48 | transmembrane protein 48 | 1.24 | 1.18E-03 |
| 8070083 | TMEM50B | transmembrane protein 50B | 1.23 | 1.84E-04 |
| 8160284 | HAUS6 | HAUS augmin-like complex, subunit 6 | 1.23 | 8.42E-04 |
| 8054329 | RNF149 | ring finger protein 149 | 1.23 | 1.05E-03 |
| 7914834 | PSMB2 | proteasome (prosome, macropain) subunit, beta type, 2 | 1.23 | 9.86E-04 |
| 8065948 | FER1L4 | fer-1-like 4 (C. elegans) pseudogene | 1.23 | 6.01E-04 |
| 8027566 | CEBPG | CCAAT/enhancer binding protein (C/EBP), gamma | 1.22 | 6.71E-04 |
| 7909782 | RRP15 | ribosomal RNA processing 15 homolog (S. cerevisiae) | 1.21 | 1.21E-03 |
| 7902382 | RABGGTB | Rab geranylgeranyltransferase, beta subunit | 1.20 | 1.36E-03 |
| 8135250 | PSMC2 | proteasome (prosome, macropain) 26S subunit, ATPase, 2 | 1.20 | 1.28E-04 |
| 8113305 | CHD1 | chromodomain helicase DNA binding protein 1 | 1.20 | 1.22E-03 |
| 7916356 | HSPB11 | heat shock protein family B (small), member 11 | 1.19 | 1.48E-04 |
| 7966462 | NAA25 | N(alpha)-acetyltransferase 25, NatB auxiliary subunit | 1.19 | 4.96E-04 |
| 8134318 | CASD1 | CAS1 domain containing 1 | 1.17 | 6.12E-04 |
| 7897460 | SLC25A33 | solute carrier family 25, member 33 | 1.17 | 1.33E-03 |
| 8060813 | MCM8 | minichromosome maintenance complex component 8 | 1.15 | 4.63E-04 |
| 8133633 | NSUN5P2 | NOP2/Sun domain family, member 5 pseudogene 2 | 1.14 | 4.90E-04 |
| 8133633 | NSUN5P1 | NOP2/Sun domain family, member 5 pseudogene 1 | 1.14 | 4.90E-04 |
| 8133633 | NSUN5 | NOP2/Sun domain family, member 5 | 1.14 | 4.90E-04 |
| 8028514 | EIF3K | eukaryotic translation initiation factor 3, subunit K | 1.11 | 1.18E-03 |
| 8020343 | ANKRD20A5 | ankyrin repeat domain 20 family, member A5 | 0.87 | 1.41E-03 |
| 8124440 | HIST1H3G | histone cluster 1, H3g | 0.76 | 7.49E-04 |
| 8086391 | CCK | cholecystokinin | 0.76 | 3.12E-04 |
| 8037079 | ATP1A3 | ATPase, Na+/K+ transporting, alpha 3 polypeptide | 0.72 | 1.06E-03 |
| 8109086 | ADRB2 | adrenergic, beta-2-, receptor, surface | 0.71 | 1.18E-03 |
| 8145736 | NRG1 | neuregulin 1 | 0.71 | 3.14E-04 |
| 8132694 | IGFBP1 | insulin-like growth factor binding protein 1 | 0.66 | 2.70E-04 |
| 8169898 | RAB33A | RAB33A, member RAS oncogene family | 0.65 | 1.22E-06 |
| 7972157 | EDNRB | endothelin receptor type B | 0.64 | 9.69E-04 |
| 8015208 | KRTAP2-2 | keratin associated protein 2-2 | 0.63 | 9.26E-04 |
| 8015206 | KRTAP2-1 | keratin associated protein 2-1 | 0.61 | 6.58E-04 |
| 8169094 | IL1RAPL2 | interleukin 1 receptor accessory protein-like 2 | 0.61 | 4.14E-04 |
| 7960771 | CD163L1 | CD163 molecule-like 1 | 0.60 | 5.03E-05 |
| 7938366 | WEE1 | WEE1 homolog (S. pombe) | 0.60 | 9.98E-04 |
| 8047467 | CDK15 | cyclin-dependent kinase 15 | 0.60 | 5.64E-06 |
| 8121225 | GRIK2 | glutamate receptor, ionotropic, kainate 2 | 0.59 | 1.90E-04 |
| 8057797 | SDPR | serum deprivation response | 0.58 | 1.16E-03 |
| 7964665 | DPY19L2 | dpy-19-like 2 (C. elegans) | 0.57 | 3.55E-05 |
| 7997726 | FOXF1 | forkhead box F1 | 0.57 | 8.96E-05 |
| 7983239 | CKMT1B | creatine kinase, mitochondrial 1B | 0.55 | 2.27E-05 |
| 7983256 | CKMT1B | creatine kinase, mitochondrial 1B | 0.55 | 2.27E-05 |
| 7983239 | CKMT1A | creatine kinase, mitochondrial 1A | 0.55 | 2.27E-05 |
| 7983256 | CKMT1A | creatine kinase, mitochondrial 1A | 0.55 | 2.27E-05 |
| 7927694 | PHYHIPL | phytanoyl-CoA 2-hydroxylase interacting protein-like | 0.54 | 7.24E-05 |
| 8117106 | RNF144B | ring finger protein 144B | 0.52 | 4.64E-04 |
| 8140668 | SEMA3A | sema domain, immunoglobulin domain (Ig), short basic domain, secreted, (semaphorin) 3A | 0.49 | 7.28E-06 |
| 8092765 | MB21D2 | Mab-21 domain containing 2 | 0.49 | 5.52E-04 |
| 7974882 | SYT16 | synaptotagmin XVI | 0.48 | 1.86E-05 |
| 8069582 | TMPRSS15 | transmembrane protease, serine 15 | 0.47 | 1.78E-05 |
| 8114991 | SH3TC2 | SH3 domain and tetratricopeptide repeats 2 | 0.47 | 3.60E-04 |
| 8106418 | CRHBP | corticotropin releasing hormone binding protein | 0.46 | 1.15E-05 |
| 8174201 | BEX1 | brain expressed, X-linked 1 | 0.43 | 3.39E-04 |
| 8150962 | TOX | thymocyte selection-associated high mobility group box | 0.43 | 4.39E-04 |
| 8015210 | LOC730755 | keratin associated protein 2-4-like | 0.42 | 3.87E-05 |
| 8019576 | LOC730755 | keratin associated protein 2-4-like | 0.42 | 3.87E-05 |
| 8015210 | KRTAP2-2 | keratin associated protein 2-2 | 0.42 | 3.87E-05 |
| 8092726 | CLDN1 | claudin 1 | 0.42 | 3.12E-05 |
| 8142981 | PODXL | podocalyxin-like | 0.40 | 7.83E-04 |
| 8105908 | OCLN | occludin | 0.36 | 2.15E-05 |
| 8138888 | PDE1C | phosphodiesterase 1C, calmodulin-dependent 70kDa | 0.34 | 1.55E-04 |
| 8104901 | IL7R | interleukin 7 receptor | 0.33 | 1.26E-04 |
| 8168678 | FAM133A | family with sequence similarity 133, member A | 0.32 | 1.64E-04 |
| 8172022 | TMEM47 | transmembrane protein 47 | 0.31 | 1.10E-04 |
| 8175531 | CDR1 | cerebellar degeneration-related protein 1, 34kDa | 0.31 | 4.48E-05 |
| 8175562 | MAGEC2 | melanoma antigen family C, 2 | 0.29 | 3.51E-05 |
| 8175543 | SPANXE | SPANX family, member E | 0.29 | 4.87E-04 |
| 8175543 | SPANXD | SPANX family, member D | 0.29 | 4.87E-04 |
| 8175543 | SPANXC | SPANX family, member C | 0.29 | 4.87E-04 |
| 8175543 | SPANXA2 | SPANX family, member A2 | 0.29 | 4.87E-04 |
| 8175543 | SPANXA1 | sperm protein associated with the nucleus, X-linked, family member A1 | 0.29 | 4.87E-04 |
| 8102468 | PRSS12 | protease, serine, 12 (neurotrypsin, motopsin) | 0.28 | 6.60E-04 |
| 8170249 | SPANXE | SPANX family, member E | 0.28 | 3.52E-04 |
| 8175550 | SPANXE | SPANX family, member E | 0.28 | 3.52E-04 |
| 8170249 | SPANXD | SPANX family, member D | 0.28 | 3.52E-04 |
| 8175550 | SPANXD | SPANX family, member D | 0.28 | 3.52E-04 |
| 8170249 | SPANXC | SPANX family, member C | 0.28 | 3.52E-04 |
| 8175550 | SPANXC | SPANX family, member C | 0.28 | 3.52E-04 |
| 8170249 | SPANXA2 | SPANX family, member A2 | 0.28 | 3.52E-04 |
| 8175550 | SPANXA2 | SPANX family, member A2 | 0.28 | 3.52E-04 |
| 8170249 | SPANXA1 | sperm protein associated with the nucleus, X-linked, family member A1 | 0.28 | 3.52E-04 |
| 8175550 | SPANXA1 | sperm protein associated with the nucleus, X-linked, family member A1 | 0.28 | 3.52E-04 |
| 8057803 | TMEFF2 | transmembrane protein with EGF-like and two follistatin-like domains 2 | 0.27 | 2.88E-05 |
| 8072626 | TIMP3 | TIMP metallopeptidase inhibitor 3 | 0.26 | 1.06E-06 |
| 7947156 | MUC15 | mucin 15, cell surface associated | 0.21 | 3.96E-05 |
| 7902441 | ST6GALNAC5 | ST6 (alpha-N-acetyl-neuraminyl-2,3-beta-galactosyl-1,3)-N-acetylgalactosaminide alpha-2,6-sialyltransferase 5 | 0.16 | 4.09E-05 |
| 8081431 | ALCAM | activated leukocyte cell adhesion molecule | 0.16 | 6.34E-06 |
| 8180266 | ST6GALNAC5 | ST6 (alpha-N-acetyl-neuraminyl-2,3-beta-galactosyl-1,3)-N-acetylgalactosaminide alpha-2,6-sialyltransferase 5 | 0.14 | 5.54E-06 |
| 8023593 | MC4R | melanocortin 4 receptor | 0.11 | 1.17E-04 |
| 8175871 | L1CAM | L1 cell adhesion molecule | 0.10 | 6.18E-05 |
| 7922343 | TNFSF4 | tumor necrosis factor (ligand) superfamily, member 4 | 0.10 | 4.43E-06 |
| 8140650 | SEMA3E | sema domain, immunoglobulin domain (Ig), short basic domain, secreted, (semaphorin) 3E | 0.09 | 7.91E-06 |
| 8021081 | SLC14A1 | solute carrier family 14 (urea transporter), member 1 (Kidd blood group) | 0.07 | 1.86E-04 |

Genes significantly upregulated or downregulated following double-IR in PG35s showing FDR < 0.05. Indicated nominal p-value was calculated with two-tailed unpaired t-test.
